# Advances in cytogenetics of Brazilian rodents: cytotaxonomy, chromosome evolution and new karyotypic data

**DOI:** 10.3897/CompCytogen.v11i4.19925

**Published:** 2017-12-21

**Authors:** Camilla Bruno Di-Nizo, Karina Rodrigues da Silva Banci, Yukie Sato-Kuwabara, Maria José de J. Silva

**Affiliations:** 1 Laboratório de Ecologia e Evolução, Instituto Butantan, Avenida Vital Brazil, 1500, CEP 05503-900, São Paulo, SP, Brazil; 2 Departamento de Genética e Biologia Evolutiva, Instituto de Biociências, Universidade de São Paulo, Rua do Matão 277, CEP 05508-900, São Paulo, SP, Brazil

**Keywords:** Chromosomes, Rodentia, karyotype evolution, Carterodon sulcidens, Neacomys

## Abstract

Rodents constitute one of the most diversified mammalian orders. Due to the morphological similarity in many of the groups, their taxonomy is controversial. Karyotype information proved to be an important tool for distinguishing some species because some of them are species-specific. Additionally, rodents can be an excellent model for chromosome evolution studies since many rearrangements have been described in this group.This work brings a review of cytogenetic data of Brazilian rodents, with information about diploid and fundamental numbers, polymorphisms, and geographical distribution. We point out that, even with the recent efforts on cytogenetic studies in this group, many species lack karyotypic data. Moreover, we describe for the first time the karyotype of Carterodon sulcidens (Lund, 1838) (Family Echimyidae), a new fundamental number for an undescribed species of Neacomys Thomas, 1900 (Family Cricetidae, Subfamily Sigmodontinae), and illustrate the karyotype of a Brazilian specimen of Mus musculus Linnaeus, 1758 (Family Muridae). This review compiles the cytogenetic data on Brazilian rodents reported in the last three decades, after the last revision published in 1984, including synonyms, chromosomal variations, and geographic distribution. Additionally, it also reinforces that Brazilian biodiversity is still poorly known, considering the new data reported here.

## Introduction

More than three decades after the last revision of cytogenetics of Brazilian rodents (Kasahara and Yonenaga-Yassuda 1984), in which the karyotypes of approximately 60 species were reported, several new karyotypes and chromosomal rearrangements have been described. In the last 30 years, huge progress has been made, and up to this date, new species have frequently been described. However, as we shall explore herein, there still remain gaps in knowledge about many species.

Cytogenetic information on Brazilian rodents was firstly described by Cestari and Imada (1968) for the species referred to as Akodon arviculoides cursor Thomas, 1913. From then on, cytogenetic data confirmed the great chromosomal variability in rodents, especially after the advent of banding techniques in the beginning of the 1970s.

Throughout the following decades, several Master dissertations and PhD theses have addressed cytogenetic studies on Brazilian rodents. It became evident that karyotypic data could contribute to accurate taxonomic information, since different names were applied to groups that shared the same karyotype, and very distinct karyotypes were attributed to a single species. Additionally, major fieldwork efforts in Brazil (especially in unexplored areas) have led to the discovery of many new species.

The increasing number of cytogenetic studies on rodents resulted in the characterization of banding patterns, recognition of sex chromosomes, identification of supernumerary chromosomes, pericentric inversions and Robertsonian rearrangements, variations in the amount and localization of constitutive heterochromatin, and recognition of species (cytotaxonomy). These discoveries have led researchers to consider that rodents have undergone a “karyotypic explosion” process and that they stand out as an excellent group for chromosomal evolution studies, since they present many examples of chromosome rearrangements. These rearrangements may have played an important role in karyotype diversification and speciation, with the reduction of gene flow due to meiotic problems (King 1993, Rieseberg 2001, Patton 2004, Faria and Navarro 2010).

Previously, chromosome evolution studies were essentially based on the comparison of banding patterns (Yonenaga-Yassuda et al. 1975, 1987a, Leal-Mesquita et al. 1992, Silva and Yonenaga-Yassuda 1999). Later, the association of cytogenetics with molecular biology allowed for a new important approach for studying karyotype evolution. Notwithstanding, molecular cytogenetics allows the localization of specific DNA sequences in the chromosomes based on DNA denaturation and its subsequent annealing with complementary sequences. In Brazilian rodents, localization of specific sequences using fluorescence *in situ* hybridization (FISH) was specifically applied in the Akodontini and Oryzomyini tribes of the Family Cricetidae, Subfamily Sigmodontinae, which is traditionally divided into 10 tribes and one *incertae sedis* group (Pardiñas et al. 2015a). Nevertheless, this kind of approach is still lacking for the other tribes of Sigmodontinae, and the remaining rodent families, mainly because of the difficulty in obtaining specific probes.

FISH was first performed using telomeric sequence probes, revealing that, besides the telomeric position itself, the sequences could also be detected at telomeric interstitial sites (ITS), such as those present in the Sigmodontinae genus Akodon Meyen, 1833, Thaptomys Thomas, 1916, and Cerradomys Weksler, Percequillo & Voss, 2006 (Fagundes et al. 1997a, Fagundes and Yonenaga-Yassuda 1998, Silva and Yonenaga-Yassuda 1998a, Andrades-Miranda et al. 2002a, Ventura et al. 2004, 2006). These ITS were correlated with components of constitutive heterochromatin, amplification of TTAGGG_n_ sequences, telomeres remnants after chromosomal rearrangements or reservoirs for future fission rearrangements. On the other hand, the absence of ITS in other Sigmodontinae species with chromosome polymorphisms, such as Oligoryzomys Bangs, 1900, and Rhipidomys Tschudi, 1845, was also described (Silva and Yonenaga-Yassuda 1997, 1999).

More recently, probes from entire chromosomes were obtained by microdissection or flow sorting, representing a breakthrough in evolutionary studies. The first Brazilian study employing this technique was published by Fagundes et al. (1997b), in which the largest pair (pair 1) of the karyotype of the rodent Akodon cursor (Winge, 1887) (Subfamily Sigmodontinae, tribe Akodontini) was obtained in order to investigate regions of homology between chromosomes of this species and Akodon montensis Thomas, 1913.

More than one decade later, Hass et al. (2008), using Mus musculus commercial chromosome probes, established chromosomal homology maps between five species of the tribe Akodontini, plus one Oryzomyini species. One year later, Ventura et al. (2009) performed chromosome painting using Akodon species-specific probes.

After the tribe Akodontini, Oryzomyini is the second most studied tribe by chromosome painting from the Subfamily Sigmodontinae. Comparisons between Hylaeamys megacephalus (G. Fischer, 1814) and Cerradomys langguthi Percequillo, Hingst-Zaher & Bonvicino, 2008 were performed by Nagamachi et al. (2013), and Di-Nizo et al. (2015) studied chromosome evolution within the genus Oligoryzomys. In addition, chromosome painting using Hylaeamys megacephalus probes was performed to compare the Akodontini and Oryzomyini tribes (Suárez et al. 2015, Pereira et al. 2016) and, more recently, two populations of Oecomys catherinae Thomas, 1909 were also evaluated (Malcher et al. 2017).

The role of cytogenetics in species recognition (cytotaxonomy) has been know for a while, considering that many rodents’ species are morphologically similar (Bonvicino and Weksler 1998, Christoff et al. 2000, Percequillo et al. 2008). In addition, molecular phylogenetics improved the possibility of recognizing monophyletic clades. In fact, the proper identification of undescribed species is only possible with the association of morphology, cytogenetics, geographic distribution and molecular phylogeny. Altogether, these different approaches are essential not only for identifying the cryptic Brazilian biodiversity but also for public health programs, since some rodents’ species are Hantavirus reservoirs (Souza et al. 2002, Lemos et al. 2004).

Therefore, the aim of this review is to compile all the cytogenetic data available for Brazilian rodents, presenting not only the diploid and fundamental numbers, but also the chromosomal polymorphisms, synonyms, and geographic distribution. In addition, we describe for the first time the karyotype of the monotypic species Carterodon sulcidens, and show the karyotype of Brazilian specimen of the introduced rodent Mus musculus for the first time. A new fundamental number for a putative undescribed species of Neacomys is also reported. In addition, to investigate phylogenetic relationships among Neacomys species, molecular analyses based on the gene cytochrome *b* were performed. This work discusses the most common rearrangements in each group, by pointing out the species which could represent complexes of species (thus needing revision) or present polymorphisms, as well as highlighting the species and families that lack cytogenetic information.

## Material and methods

### Literature revision

This review was done after an extensive revision of the literature, including Master’s and Ph.D. theses, when available (Table 1). Abstracts from congresses and conferences were not considered, since karyotype pictures were only available during the events and access to this kind of material is restricted. Chromosome rearrangements in Table 1 were named as described in the literature (for example Robertsonian rearrangement, centric fusion, etc.). However, in the text, we refer to centric fusion/fission as a synonym of Robertsonian rearrangement (Sumner 2003). Except for the species that have not been formally described (e.g. Thaptomys sp., Proechimys gr. goeldii, etc.), the taxonomical classification follows the one proposed by Patton et al. (2015) and Fabre et al. (2016), that recently included Myocastor Kerr, 1792 within the Family Echimyidae.

**Table 1.** Compilation of cytogenetic data of Brazilian rodents, with the respective synonyms, diploid number (2n) and fundamental number (FN), karyotypic variation, localities (according to Bonvicino et al. 2008 and Patton et al. 2015) and references.

		Species	Synonyms	2n	FN	Karyotypic Variations	Distribution	References
**Family Caviidae**		Cavia aperea Erxleben, 1777	-	64	116, 124	-	PE, SE, AL, BA, MG, GO, MT, MS, MG, SP, PR and SC	Maia 1984, Bonvicino et al. 2008, Gava et al. 2011
		Cavia fulgida Wagler, 1831	-	64	124	-	Eastern Brazil, between MG and SC	Woods and Kilpatrick 2005, Walker et al. 2014
		Cavia intermedia Cherem, Olimpio, and Ximénez, 1999	Cavia aff. magna	62	108	-	Endemic from SC (Ilhas Moleques do Sul)	Gava et al. 1998, Woods and Kilpatrick 2005
		Cavia magna Ximénez, 1980	-	62; 64	102; 124	Pericentric inversions; addition and deletion of constitutive hetechromatin; Robertsonian rearrangement	RS and SC	Bonvicino et al. 2008, Gava et al. 2011, Walker et al. 2014
		Cavia porcellus (Linnaeus, 1758)	-	64*	100-102	Polymorphism in chromosome 1	All Brazilian States	Bonvicino et al. 2008, Walker et al. 2014
		Galea flavidens (Brandt, 1835)	-	N/A	N/A	-	Northwestern MG and Northeastern GO	Bonvicino et al. 2008
		Galea spixii (Wagler, 1831)	-	64	118	-	PA, MT, MG, BA, PE, PB, RN, CE, PI, MA and DF	Maia 1984, Bonvicino et al. 2008
		Hydrochoerus hydrochaeris (Linnaeus, 1766)	-	66	102	-	All Brazilian States, except CE	Wurster et al. 1971, Bonvicino et al. 2008
		Kerodon acrobata Moojen, 1997	-	52	92	-	Northeastern GO	Bonvicino et al. 2008, Zappes et al. 2014
		Kerodon rupestris (Wied-Neuwied, 1820)	-	52	92, 94	Pericentric inversion	From PI and CE to Northern MG	Maia 1984, Bonvicino et al. 2008, Lessa et al. 2013
**Family Cricetidae - Subfamily Sigmodontinae**	**Tribe Akodontini**	Akodon azarae (J. B. Fischer, 1829)	-	37-38	40-44	Variation in the Y morphology; deletion of the X long arm	Southern Brazil	Kasahara and Yonenaga-Yassuda 1984, Vitullo et al. 1986, Sbalqueiro 1989, Pardiñas et al. 2015b
		Akodon cursor (Winge, 1887)	Akodon arviculoides	14-16	18-26	Pericentric inversions in pairs 2, 4 and 6; centric fusion and pericentric inversion in pairs 1 and 3; trisomy of the pair 7; ITS	Atlantic Forest formations in Eastern Brazil from PB to PR and Eastern MG	Cestari and Imada 1968, Fagundes et al. 1997a, Fagundes et al. 1997b, Fagundes et al. 1998, Geise et al. 1998
		Akodon lindberghi Hershkovitz, 1990	Akodon sp.	42	42	ITS	Cerrado habitat, Central and Southeastern Brazil	Svartman 1989, Svartman and Almeida 1994, Geise 1995
		Akodon montensis Thomas, 1913	Akodon aff. arviculoides, Akodon sp.	23; 24-26; 24/25; 23/24	40; 42; 44	X monosomy; 1 or 2 B chromosomes; mosaicism; reciprocal translocation (1, 6); sex chromosome heteromorphism	Southeastern Brazil, from RJ to RS, including gallery Forest settings in MG and GO	Geise et al. 1998, Fagundes et al. 1997b, Fagundes et al. 2000
**Family Cricetidae - Subfamily Sigmodontinae**	**Tribe Akodontini**	Akodon mystax Hershkovitz, 1998	-	42, 44	42	-	Pico da Bandeira, in the border of MG and ES	Musser and Carleton 2005, Gonçalves et al. 2007, Pardiñas et al. 2015b
		Akodon paranaensis Christoff, Fagundes, Sbalqueiro, Mattevi and Yonenaga- Yassuda, 2000	Akodon serrensis	44	44	Non-disjunction of the sex chromosomes (2n = 43 and 45)	Eastern RJ and SP and Southern Brazil	Christoff et al. 2000
		Akodon reigi E. M. González, Langguth & Oliveira, 1998	-	44	44	-	Southernmost Brazil (RS)	Musser and Carleton 2005
		Akodon sanctipaulensis Hershkovitz, 1990	-	N/A	N/A	-	Serra do Mar, Southeastern Brazil	Musser and Carleton 2005
		Akodon sp. n.	-	9; 10	14-16	X monosomy; pericentric inversion in pair 3; ITS	Only known from its type locality, MT	Silva and Yonenaga-Yassuda 1998a
		Akodon toba Thomas, 1921	Akodon varius	40*; 42-43*	40*; 44*	Karyotype of specimens from Paraguay	Southwestern MS	Bonvicino et al. 2008, Pardinãs et al. 2015a
		Bibimys labiosus (Winge, 1887)	-	70	80	-	Northern RS, and Southeastern MG and RJ	Bonvicino et al. 2008, Gonçalves et al. 2007
		Blarinomys breviceps (Winge, 1887)	-	28; 31 (29+2Bs); 34; 37 (36 + 1B); 43 (39 + 4Bs); 45 (44 + 1B); 52; 52 (50 + 2Bs)	48, 50; 50; 50; 50; 50; 50, 51; 50; 50	B chromosomes; Robertsonian rearrangement; ITS	Atlantic Forest regions of Southeastern Brazil (from BA to SP, and Eastern MG)	Silva et al. 2003, Musser and Carleton 2005, Geise et al. 2008, Ventura et al. 2012
		Brucepattersonius griserufescens Hershkovitz, 1998	-	52	52, 53	Pericentric inversion in pair 2	Eastern MG, and ES to RJ	Bonvicino et al. 1998a, Musser and Carleton 2005
		Brucepattersonius igniventris Hershkovitz, 1998	-	N/A	N/A	-	Southeastern SP	Musser and Carleton 2005, Bonvicino et al. 2008, Rossi 2011
		Brucepattersonius iheringi (Thomas, 1896)	Oxymycterus iheringi	52	52	-	Southern Brazil	Musser and Carleton 2005, Vilela 2005
		Brucepattersonius soricinus Hershkovitz, 1998	-	52	52	-	Eastern SP and PR	Musser and Carleton 2005, Di-Nizo et al. 2014
		Castoria angustidens (Thomas, 1902)	Akodon sp., A. leucogula, A. serrensis	46	46	ITS	Atlantic Forest from Southeastern ES to RS	Geise et al. 1998, Christoff et al. 2000, Abreu et al. 2014, Pardiñas et al. 2015b, Pardiñas et al. 2016a
		Deltamys araucaria Quintela, Bertuol, González, Cordeiro-Estrela, Freitas, Gonçalves, 2017	-	34	34	-	Only known from its type locality, São Francisco de Paula/RS	Quintela et al. 2017
		Deltamys kempi Thomas, 1917	-	35-38	38	Centric fusion/fission; multiple sex determination system.	Eastern RS	Sbalqueiro et al. 1984, Castro et al. 1991, Musser and Carleton 2005, Bonvicino et al. 2008
**Family Cricetidae - Subfamily Sigmodontinae**	**Tribe Akodontini**	Deltamys sp.	-	40	40	-	Esmeralda (RS)	Ventura et al. 2011
		Gyldenstolpia fronto Winge, 1887	Kunsia fronto	N/A	N/A	-	Lagoa Santa (MG)	Musser and Carleton 2005, Pardiñas et al. 2008, Pardiñas and Bezerra 2015
		Gyldenstolpia planaltensis (Avila-Pires, 1972)	Kunsia fronto planaltensis	N/A	N/A	-	Westcentral Brazil	Pardiñas and Bezerra 2015
		Juscelinomys candango Moojen, 1965	-	N/A	N/A	-	DF	Musser and Carleton 2005
		Kunsia tormentosus (Lichtenstein, 1830)	-	44	42	-	Westcentral Brazil	Andrades-Miranda et al. 1999, Musser and Carleton 2005
		Necromys lasiurus (Lund, 1840)	Zygodontomys lasiurus, Bolomys lasiurus	34, 33, 33/34	34	Robertsonian rearrangement; centric fusion, X polymorphism; mosaicism (XX/X0)	Southern Amazon River, Brazil	Maia and Langguth 1981, Kasahara and Yonenaga-Yassuda 1983, Svartman and Almeida 1993a, Musser and Carleton 2005
		Necromys urichi (J. A. Allen & Chapman, 1897)	-	18	30	-	Northern Brazil	Reig et al. 1986, Musser and Carleton 2005
		Oxymycterus amazonicus Hershkovitz, 1994	-	54	N/A	-	Lower Amazon Basin, Southern Amazon River, between Tocantins and Madeira Rivers, Central Brazil, Northwestern MT	Bonvicino et al. 1998a, Musser and Carleton 2005
		Oxymycterus caparoae Hershkovitz, 1998	-	54	64	-	Eastern MG and ES to RJ	Bonvicino et al. 1998a, Musser and Carleton 2005
		Oxymycterus dasytrichus (Schinz, 1821)	Oxymycterus angularis, Oryzomys hispidus, Oryzomys roberti	54	64	-	Atlantic and interior forest of Eastern Brazil (PE, AL, SE, BA, MG, ES, RJ, SP and PA)	Musser and Carleton 2005, Moreira et al. 2009
		Oxymycterus delator Thomas, 1903	Oxymycterus sp., Oxymycterus roberti	54	62	-	Southcentral Brazil	Svartman and Almeida 1993b , Bonvicino et al. 2005a, Musser and Carleton 2005
		Oxymycterus inca Thomas, 1900	-	54	N/A	-	Acre	Bonvicino et al. 1998a, Bonvicino et al. 2008
		Oxymycterus nasutus (Waterhouse, 1837)	-	54	64	-	Eastern RS to Eastern SP	Musser and Carleton 2005, Quintela et al. 2012
		Oxymycterus quaestor Thomas, 1903	Oxymycterus judex	54	N/A	-	Eastern Brazil, from RS to SP, and Serra dos Órgãos (RJ)	Bonvicino et al. 1998a, Bonvicino et al. 2008, Oliveira and Gonçalves 2015
		Oxymycterus rufus (G. Fischer, 1814)	-	54	66	-	Southeastern MG	Geise 1995
		Podoxymys roraimae Anthony, 1929	-	16	26	-	RR	Pérez-Zapata et al. 1992, Musser and Carleton 2005, Bonvicino et al. 2008
**Family Cricetidae - Subfamily Sigmodontinae**	**Tribe Akodontini**	Scapteromys aquaticus Thomas, 1920	-	32	40	-	Westernmost RS	Bonvicino et al. 2013
		Scapteromys meridionalis Quintela, Gonçalves, Althoff, Sbalqueiro, Oliveira, Freitas, 2014	Scapteromys sp. 1, Scapteromys sp. 2	34, 36	40	Centric fusion	Southern Brazil	Freitas et al. 1984, Bonvicino et al. 2013, Quintela et al. 2014
		Scapteromys tumidus (Waterhouse, 1837)	-	24	40	-	Southernmost Brazil (RS)	Brum-Zorrilla et al. 1986, Musser and Carleton 2005
		Thalpomys cerradensis Hershkovitz, 1990	-	36	34	-	Cerrado of Central Brazil	Andrade et al. 2004, Musser and Carleton 2005
		Thalpomys lasiotis Thomas, 1916	Akodon reinhardti	37, 38	38	Centric fusion/fission; heterochromatin variation in an autosomal pair	Cerrado of Central Brazil	Yonenaga-Yassuda et al. 1987b
		Thaptomys nigrita (Lichtenstein, 1829)	Akodon (Thaptomys) nigrita	52	52	-	Southeastern Brazil, BA to RS	Yonenaga 1972, Yonenaga 1975, Souza 1981, Castro 1989, Fagundes 1993, Geise 1995
		Thaptomys sp.	-	50	48	ITS	Only known from its type locality - BA	Ventura et al. 2004
	**Tribe Ichthyomyini**	Neusticomys ferreirai Percequillo, Carmignotto & Silva, 2005	-	92	98	-	Amazonian lowland of MT and PA	Percequillo et al. 2005
		Neusticomys oyapocki (Dubost & Petter, 1979)	-	N/A	N/A	-	Amazonian of Northern Brazil (AP and PA)	Voss 2015a
	**Tribe Oryzomyini**	Cerradomys akroai Bonvicino, Casado & Weksler, 2014	-	60	74	-	TO	Bonvicino et al. 2014
		Cerradomys goytaca Tavares, Pessôa & Gonçalves, 2011	-	54	62, 63, 66	Different interpretation of morphology of small pairs and pericentric inversion in small chromosome	Northeastern littoral of RJ and Southern littoral of ES (Restinga region)	Tavares et al. 2011, Bonvicino et al. 2014
		Cerradomys langguthi Percequillo, Hingst- Zaher, and Bonvicino, 2008	Oryzomys sp. B	46, 48, 49, 50	56	Centric fusion/ fission; Y polymorphism; ITS	PE, MA, PB and CE	Maia and Hulak 1981, Percequillo et al. 2008, Nagamachi et al. 2013
		Cerradomys maracajuensis (Langguth & Bonvicino, 2002)	-	56	58	-	Central MT and MS	Langguth and Bonvicino 2002, Bonvicino et al. 2008, Bonvicino et al. 2014
		Cerradomys marinhus (Bonvicino, 2003)	-	56	54	-	GO and Southeatern BA	Bonvicino 2003, Bonvicino et al. 2008
		Cerradomys scotti (Langguth & Bonvicino, 2002)	Oryzomys gr. subflavus	58	70-72	Pericentric inversion in small chromosome pair; X and Y polymorphisms	GO, Southern MT, Southeastern RO, Northern MS, Western MG and BA, Southeastern TO and Southern PI	Langguth and Bonvicino 2002, Bonvicino et al. 2008
**Family Cricetidae - Subfamily Sigmodontinae**	**Tribe Oryzomyini**	Cerradomys subflavus (Wagner, 1842)	-	54; 55; 56	62; 63; 64	Robertsonian rearrangement; pericentric inversion in pair 5; X and Y polymorphisms; ITS	PB, PE, AL, BA, MG and SP	Almeida and Yonenaga-Yassuda 1985, Bonvicino et al. 2008
		Cerradomys vivoi Percequillo, Hingst- Zaher & Bonvicino, 2008	Oryzomys gr. subflavus	50	62, 63	Pericentric inversion; ITS	MG, BA and SE	Andrades-Miranda et al. 2002a, Percequillo et al. 2008
		Drymoreomys albimaculatus Percequillo, Weksler & Costa, 2011	-	62	62	ITS	Atlantic Forest of SP	Percequillo et al. 2011, Suárez-Villota et al. 2013
		Euryoryzomys emmonsae (Musser, Carleton, Brothers & Gardner, 1998)	Oryzomys emmonsae	80	86	-	Centraleastern PA	Musser et al. 1998, Bonvicino et al. 2008
		Euryoryzomys lamia (Thomas, 1901)	-	58; 60, 64	82, 84; 84	One name with different karyotypes associated	Western MG and Eastern GO	Bonvicino et al. 1998b, Andrades-Miranda et al. 2000, Bonvicino et al. 2008
		Euryoryzomys macconnelli (Thomas, 1910)	Oryzomys macconnelli	64; 58	70; 90	One name with different karyotypes associated	Northern Brazil	Patton et al. 2000, Bonvicino et al. 2008
		Euryoryzomys nitidus (Thomas, 1884)	Oryzomys nitidus	80	86	-	AC, RO, Western MT and Southern AM	Patton et al. 2000, Bonvicino et al. 2008
		Euryoryzomys russatus (Wagner, 1848)	Oryzomys capito, Oryzomys nitidus, *O. intermedius*, Oryzomys russatus	80; 80/81	86	Dissociation of the X chromosome; X and Y polymorphisms	Southeastern Brazil from BA to RS	Yonenaga et al. 1976, Almeida 1980, Zanchin 1988, Silva 1994, Geise 1995, Musser and Carleton 2005, Bonvicino et al. 2008
		Euryoryzomys sp.	-	76	86	-	Only known from its type locality - CE	Silva et al. 2000
		Holochilus brasiliensis (Desmarest, 1819)	-	55; 56-58	56	Centric fusion; 0 to 2 B chromosomes	Southern and Southeastern Brazil	Freitas et al. 1983, Yonenaga-Yassuda et al. 1987a, Bonvicino et al. 2008
		Holochilus chacarius Thomas, 1906	-	48-56*	56-60*	Centric fusion, inversion and B chromosomes	Western MS	Vidal et al. 1976, Bonvicino et al. 2008, Gonçalves et al. 2015
		Holochilus sciureus Wagner, 1842	Holochilus brasiliensis	55-56	56	Centric fusion and heteromorphism in pair 1	Northern, Northeastern and Central Brazil	Freitas et al. 1983, Patton et al. 2000, Bonvicino et al. 2008
		Holochilus vulpinus (Brants, 1827)	Holochilus brasiliensis vulpinus	40	56	-	Western RS	Freitas et al. 1983, Bonvicino et al. 2008
		Hylaeamys laticeps (Lund, 1840)	Oryzomys capito, O. c. laticeps, Oryzomys megacephalus, Hylaeamys laticeps	48	60	-	Eastern Atlantic Forest, from BA to Northern RJ	Percequillo 2015b
**Family Cricetidae - Subfamily Sigmodontinae**	**Tribe Oryzomyini**	Hylaeamys megacephalus (G. Fischer, 1814)	Oryzomys capito, O. c. laticeps, Oryzomys megacephalus;	54	62	-	Northern and Central Brazil	Musser et al. 1998, Patton et al. 2000, Musser and Carleton 2005
		Hylaeamys oniscus (Thomas, 1904)	Oryzomys capito oniscus	52	62	-	Northern Rio São Francisco, in PB, PE and AL	Maia 1990, Brennand et al. 2013
		Hylaeamys perenensis (J. A. Allen, 1901)	Oryzomys perenensis	52	62	-	Western Brazil	Patton et al. 2000, Bonvicino et al. 2008
		Hylaeamys seuanezi (Weksler, Geise & Cerqueira, 1999)	Oryzomys capito, O. c. oniscus, Oryzomys laticeps	48	60	-	Southern Rio São Francisco, from BA to RJ	Brennand et al. 2013
		Hylaeamys yunganus (Thomas, 1902)	Oryzomys yunganus	52-60	62-67	Chromosome polymorphisms within and between western and eastern population	Northern Brazil	Musser et al. 1998, Patton et al. 2000, Bonvicino et al. 2008
		Lundomys molitor (Winge, 1887)	Holochilus magnus	52	58	Variation in the X chromosome	Central RS	Freitas 1980, Freitas et al. 1983, Bonvicino et al. 2008
		Microakodontomys transitorius Hershkovitz, 1993	-	38	46	-	DF	Musser and Carleton 2005, Bonvicino et al. 2008, Paresque and Hanson 2015
		Neacomys amoenus amoenus Thomas, 1903	Neacomys spinosos amoenus	64	68	-	Northwestern Brazil	Patton et al. 2000, Bonvicino et al. 2008, Hurtado and Pacheco 2017
		Neacomys dubosti Voss, Lunde & Simmons, 2001	-	62, 64	68	Robertsonian rearrangement	Northern AP	Voss et al. 2001, Musser and Carleton 2005, Bonvicino et al. 2008, Silva et al. 2015
		Neacomys guianae Thomas, 1905	-	56	N/A	-	Northern Brazil	Musser and Carleton 2005, Silva et al. 2015
		Neacomys minutus Patton, da Silva & Malcolm, 2000	-	35-36	40	Robertsonian rearrangement	Southwestern AM	Patton et al. 2000, Bonvicino et al. 2008
		Neacomys musseri Patton, da Silva & Malcolm, 2000	-	34	64-68	Pericentric inversion	Westernmost AC	Patton et al. 2000, Musser and Carleton 2005, Bonvicino et al. 2008
		Neacomys paracou Voss, Lunde & Simmons, 2001	-	56	62, 66	Pericentric inversion	Northernmost Brazil	Voss et al. 2001, Bonvicino et al. 2008, Silva et al. 2015
		Neacomys sp.	-	58	64, 66, 70	Differences in amount of heterochromatin, pericentric inversion	PA and MT	Silva et al. 2015, present study
		Nectomys apicalis Peters, 1861	-	42	40	-	Westernmost Brazil, AC and AM	Patton et al. 2000, Musser and Carleton 2005
		Nectomys rattus Pelzeln, 1883	Nectomys squamipes, N. mattensis	52-55	52, 54, 56	B chromosomes; X and Y polymorphisms	Northern, Northeastern and Central Brazil	Furtado 1981, Maia et al. 1984, Yonenaga-Yassuda et al. 1988, Zanchin 1988, Svartman 1989, Bonvicino 1994, Bonvicino et al. 1996, Silva and Yonenaga-Yassuda 1998b, Silva 1999, Lima-Rosa et al. 2000, Patton et al. 2000, Bonvicino et al. 2008
**Family Cricetidae - Subfamily Sigmodontinae**	**Tribe Oryzomyini**	Nectomys squamipes Brants, 1827	-	56-59; 55; 56/57	56-58; 60; 62	B chromosomes; fusion/fission of autosomes; X monossomy; X and Y polymorphisms	Southeastern Brazil from PE to Northern RS	Yonenaga 1972, Yonenaga et al. 1976, Freitas 1980, Furtado 1981, Maia et al. 1984, Yonenaga-Yassuda et al. 1988, Zanchin 1988, Silva 1994, Geise 1995, Bonvicino et al. 1996, Silva 1999, Bonvicino et al. 2008
		Oecomys auyantepui Tate, 1939	-	64; 66; 72	110; 114; 80	One name with different karyotypes associated	Northern AP and PA	Bonvicino et al. 2008, Lira 2012, Gomes Jr. et al. 2016
		Oecomys bahiensis (Hershkovitz, 1960)	Oecomys concolor bahiensis	60	62	-	BA, PE (uncertain distribution)	Langguth et al. 2005, Flores 2010, Gomes Jr. et al. 2016
		Oecomys bicolor (Tomes, 1860)	-	80	140; 142	-	Northern and Central Brazil	Suárez-Villota et al. 2017
		Oecomys catherinae Thomas, 1909	-	60	62; 64	-	Atlantic forest from PB to SC, and Cerrado and Caatinga regions of BA, GO and MG	Musser and Carleton 2005, Bonvicino et al. 2008, Suárez-Villota et al. 2017
		Oecomys cleberi Locks, 1981	-	80; 82	124, 134, 140, 142; 116	One name with different karyotypes associated	DF, PN Emas (GO), and São Joaquim da Barra and Guará (SP)	Lira 2012, Suárez-Villota et al. 2017
		Oecomys concolor (Wagner, 1845)	Oryzomys (Oecomys) concolor	60	62	-	Northwestern Brazil	Furtado 1981, Svartman 1989, Lima-Rosa et al. 2000, Musser and Carleton 2005
		Oecomys franciscorum Pardiñas, Teta, Salazar-Bravo, Myers & Galliari, 2016	-	72	90	-	Pantanal	Pardiñas et al. 2016b, Suárez-Villota et al. 2017
		Oecomys mamorae (Thomas, 1906)	-	N/A	N/A	-	Westcentral Brazil	Musser and Carleton 2005, Suárez-Villota et al. 2017
		Oecomys paricola (Thomas, 1904)	-	68; 70	72; 72, 74, 76	One name with different karyotypes associated	Central Brazil, Southern Amazon River	Musser and Carleton 2005, Suárez-Villota et al. 2017
		Oecomys rex Thomas, 1910	-	62	80	-	Northern Amazon Rio (AP and AM)	Musser and Carleton 2005, Lira 2012, Gomes Jr. et al. 2016
		Oecomys roberti (Thomas, 1904)	-	80; 82	114; 106	-	Amazon region of AM, RO and MT	Musser and Carleton 2005, Patton et al. 2000, Suárez-Villota et al. 2017
		Oecomys rutilus Anthony, 1921	-	54	82, 90	-	Eastern AM	Voss et al. 2001, Gomes Jr. et al. 2016
		Oecomys superans Thomas, 1911	-	80	108	-	Western AM	Patton et al. 2000
		Oecomys trinitatis (J. A. Allen & Chapman, 1893)	-	58	96	-	Northern AC, AM and RR, and Northwestern PA	Bonvicino et al. 2008
		Oecomys sp.	-	86	98	-	AM	Patton et al. 2000, Suárez-Villota et al. 2017
		Oecomys sp.	Oecomys cf. bicolor	80	124	-	MT	Lima-Rosa et al. 2000, Andrades-Miranda et al. 2001a
		Oecomys sp. 1	-	54	54	-	MT	Suárez-Villota et al. 2017
**Family Cricetidae - Subfamily Sigmodontinae**	**Tribe Oryzomyini**	Oecomys sp. 2	-	60	62	-	Aripuanã (MT)	Suárez-Villota et al. 2017
		Oecomys sp. 3	-	60	62	-	São Joaquim da Barra (SP)	Suárez-Villota et al. 2017
		Oecomys sp. 4	-	62	62	-	Vila Rica (MT), Parauapebas (PA)	Suárez-Villota et al. 2017
		Oligoryzomys chacoensis (Myers & Carleton, 1981)	-	58	74	-	Centraleastern Brazil	Myers and Carleton 1981, Bonvicino and Geise 2006
		Oligoryzomys flavescens (Waterhouse, 1837)	-	64-68	66-72	1 to 4 B chromosomes; sex chromosome polymorphisms	Eastern Brazil, from BA to RS	Sbalqueiro et al. 1991, Bonvicino et al. 2008, Di-Nizo 2013
		Oligoryzomys mattogrossae (J. A. Allen, 1916)	Oligoryzomys eliurus, *O. fornesi*	62	64-66	Pericentric inversion in small acrocentric pair	DF, Northern MG, GO, BA and Western PE	Bonvicino and Weksler 1998, Andrades-Miranda et al. 2001a, Bonvicino et al. 2008
		Oligoryzomys messorius (Thomas, 1901)	-	66	74	-	Northern Brazil (RO)	Andrades-Miranda et al. 2001a, Weksler and Bonvicino 2015
		Oligoryzomys microtis (J. A. Allen, 1916)	-	64	64, 66	Pericentric inversion in pair 1; X polymorphism	Amazon Basin of Brazil	Aniskin and Voloboeuv 1999, Patton et al. 2000, Musser and Carleton 2005, Di-Nizo et al. 2015
		Oligoryzomys moojeni Weksler & Bonvicino, 2005	Oligoryzomys sp.	70	72, 74, 76	Pericentric inversion in small acrocentric pairs; sex chromosome polymorphisms	Southern TO, Northern GO, e Northwestern MG	Lima et al. 2003, Weksler and Bonvicino 2005, Bonvicino et al. 2008, Di-Nizo 2013
		Oligoryzomys nigripes (Olfers, 1818)	Oligoryzomys delticola, Oryzomys eliurus	61, 62	78-82	Pericentric inversions in pairs 2, 3, 4 and 8; Sex chromosome polymorphism; mosaicism (XX/X0)	PB to Northern RS, MG and DF	Almeida and Yonenaga-Yassuda 1991, Paresque et al. 2007, Bonvicino et al. 2008, Di-Nizo 2013
		Oligoryzomys rupestris Weksler & Bonvicino, 2005	Oligoryzomys sp. 1	46	52	-	high altitudes in GO and BA	Silva and Yonenaga-Yassuda 1997, Weksler and Bonvicino 2005
		Oligoryzomys stramineus Bonvicino and Weksler, 1998	-	52	68-70	Pericentric inversion in one small acrocentric pair	Cerrado (GO and MG) and Caatinga (PB, PI e PE)	Bonvicino and Weksler 1998, Weksler and Bonvicino 2005
		Oligoryzomys utiaritensis J. A. Allen, 1916	Oligoryzomys nigripes	72	76	-	MT and PA (Transition of Cerrado and Amazon)	Agrellos et al. 2012
		Oligoryzomys sp.	Oligoryzomys cf. messorius	56	58	-	AP	Andrades-Miranda et al. 2001a, Miranda et al. 2008, Weksler and Bonvicino 2015
		Oligoryzomys sp. 2	-	44; 45	52; 53	Mosaicism of a small acrocentric pair; X chromosome polymorphisms	Only known from its type locality (Serra do Cipó, MG)	Silva and Yonenaga-Yassuda 1997
		Pseudoryzomys simplex (Winge, 1887)	-	56	54; 55	Addition of constitutive heterochromatin in pair 17	Central Brazil (MT, TO, GO, MG, SP, BA, AL and PE)	Bonvicino et al. 2008, Moreira et al. 2013
		Scolomys ucayalensis Pacheco, 1991	Scolomys juruaense	50	68	-	Westernmost Brazil (AC and AM)	Patton and da Silva 1995, Musser and Carleton 2005, Patton 2015
**Family Cricetidae - Subfamily Sigmodontinae**	**Tribe Oryzomyini**	Sooretamys angouya (G. Fischer, 1814)	-	57-60	60-64	0-2 B chromosomes	Southeastern Brazil, from ES to RS	Almeida 1980, Zanchin 1988, Silva 1994, Geise 1995, Musser and Carleton 2005, Bonvicino et al. 2008
		Zygodontomys brevicauda (J. A. Allen & Chapman, 1893)	-	86; 84; 82	96-100; 96-98; 94	One name with different karyotypes associated	Northernmost Brazil (AM, RR, PA and AP)	Mattevi et al. 2002, Bonvicino et al. 2009, Voss 2015b
	**Tribe Phyllotini**	Calassomys apicalis Pardiñas, Lessa, Salazar-Bravo & Câmara, 2014	-	62	116	-	Only known in three localities in Central MG	Pardiñas et al. 2014
		Calomys aff. expulsus	-	64	66	-	GO	Mattevi et al. 2005
		Calomys callidus (Thomas, 1916)	-	48	66	-	Western Brazil (RO to MT)	Mattevi et al. 2005, Bonvicino et al. 2010
		Calomys callosus (Rengger, 1830)	-	50	66	-	Western MS	Bonvicino et al. 2008, Bonvicino et al. 2010
		Calomys cerqueirai Bonvicino, Oliveira & Gentile, 2010	-	36; 38	66	Centric Fusion	MG and ES	Bonvicino et al. 2010, Colombi and Fagundes 2014
		Calomys expulsus (Lund, 1840)	-	66	68	-	Caatinga and Cerrado formations from PE to GO	Musser and Carleton 2005, Bonvicino and Almeida 2000
		Calomys laucha (G. Fisher, 1814)	-	64	68	-	Southermost RS	Bonvicino et al. 2008, Mattevi et al. 2005
		Calomys tener (Winge, 1887)	-	64; 66	64; 66	One name with different karyotypes associated	Atlantic Forest region and habitats bordering the Cerrado, Southeastern Brazil (GO, MG, ES, SP, BA and DF)	Bonvicino and Almeida 2000, Mattevi et al. 2005, Musser and Carleton 2005, Bonvicino et al. 2008, Salazar-Bravo 2015
		Calomys tocantinsi Bonvicino, Lima & Almeida, 2003	Calomys sp.	46	66	-	Cerrado habitats MT, TO and GO	Bonvicino et al. 2003a, Musser and Carleton 2005, Bonvicino et al. 2008
	**Tribe Reithrodontini**	Reithrodon typicus Waterhouse, 1837	-	28	40	-	Boundary between RS and Uruguay	Freitas et al. 1983, Pardiñas et al. 2015c
	**Tribe Sigmodontini**	Sigmodon alstoni (Thomas, 1881)	-	78, 80, 82*	N/A	Robertsonian polymorphisms; Karyotype of specimens from Venezuela	Northernmost Brazil (RR, AP and PA)	Voss 1992, Bonvicino et al. 2008
	**Tribe Thomasomyini**	Rhagomys rufescens (Thomas, 1886)	-	36	50	-	RJ, SP and MG	Bonvicino et al. 2008, Testoni et al. 2010
**Family Cricetidae - Subfamily Sigmodontinae**	**Tribe Thomasomyini**	Rhipidomys cariri Tribe, 2005	R. cariri baturiteensis	44	48, 50	FN=50 (type locality), FN=48 (R. cariri baturiteensis)	CE, PE and BA	Tribe 2005, Bonvicino et al. 2008, Thomazini 2009,Carvalho et al. 2012, Geise et al. 2010
		Rhipidomys emiliae (J. A. Allen, 1916)	-	44	46, 52, 64	Pericentric inversion	Eastern PA, MT (Serra do Roncador) and Western MA	Silva and Yonenaga-Yassuda 1999, Bonvicino et al. 2008, Tribe 2015
		Rhipidomys gardneri Patton, da Silva & Malcolm, 2000	-	44	50	-	Northwestern AC	Patton et al. 2000, Bonvicino et al. 2008
		Rhipidomys ipukensis R. G. Rocha, Costa & Costa, 2011	-	N/A	N/A	-	Endemic to the Araguaia-Tocantins basin	Rocha et al. 2011, Tribe 2015
		Rhipidomys itoan B. M. de A. Costa, Geise, Pereira and L. P. Costa, 2011	-	44	48-50	Pericentric inversion	RJ and Eastern SP to Southern Serra da Mantiqueira	Costa et al. 2011
		Rhipidomys leucodactylus (Tschudi, 1845)	-	44	46, 48, 52	Pericentric inversion	Northwestern Brazil (AM, AC, MT, RO, RR, AP and PA)	Zanchin et al. 1992, Silva and Yonenaga-Yassuda 1999, Patton et al. 2000, Bonvicino et al. 2008, Tribe 2015
		Rhipidomys macconnelli de Winton, 1900	-	44*	50*	Karyotype of specimens from Venezuela	AM (Serra da Neblina) and Western RR, above 1.000m of altitude	Aguilera et al. 1994, Bonvicino et al. 2008
		Rhipidomys macrurus (P. Gervais, 1855)	-	44	48-52	Pericentric inversion	Cerrado and Caatinga biomes, from CE to MT, and MG	Zanchin et al. 1992, Silva and Yonenaga-Yassuda 1999, Musser and Carleton 2005, Carvalho et al. 2012
		Rhipidomys mastacalis (Lund, 1840)	-	44	70, 74, 76, 80	Pericentric inversion	Atlantic Forest region, from PE to PR	Zanchin et al. 1992, Andrades-Miranda et al. 2002b, Paresque et al. 2004, Musser and Carleton 2005, Sousa 2005, Bonvicino et al. 2008, Carvalho et al. 2012, Tribe 2015
		Rhipidomys nitela Thomas, 1901	Rhipidomys sp. B	48; 50	68; 71, 72	Pericentric inversion in pair 8, addition and deletion of constitutive hetechromatin	Northcentral Brazil (AM, MT, AP, RR, PA, TO and GO)	Silva and Yonenaga-Yassuda 1999, Andrades-Miranda et al. 2002b, Tribe 2015
		Rhipidomys tribei B. M. de A. Costa, Geise, Pereira and L. P. Costa, 2011	-	44	50	-	Serra do Caraça, Southern MG	Zanchin et al. 1992, Costa et al. 2011
		Rhipidomys wetzeli A. L. Gardner, 1990	-	N/A	N/A	-	Northern Brazil	Fonseca et al. 1996, Tribe 2015
	**Tribe Wiedomyini**	Wiedomys cerradensis P. R. Gonçalves, Almeida & Bonvicino, 2005	-	60	88	-	Only known from its type locality (Southwestern BA)	Gonçalves et al. 2005
		Wiedomys pyrrhorhinos (Wied-Neuwied, 1821)	-	62	86, 90, 104	Pericentric inversion in the smallest pairs	Southern CE, Southeastern PI, and Western PB, PE, AL, BA and Northern MG	Maia and Langguth 1987, Gonçalves et al. 2005, Bonvicino et al. 2008, Souza et al. 2011
**Family Cricetidae - Subfamily Sigmodontinae**	***Incertae sedis***	Abrawayaomys ruschii F. Cunha & Cruz, 1979	-	58	N/A	-	ES, RJ, SP, MG and SC	Bonvicino et al. 2008, Pereira et al. 2008
		Delomys altimontanus Gonçalves & Oliveira, 2014	-	82	86	-	Disjunction distribution in Itatiaia (RJ) and Caparaó (MG)	Gonçalves and Oliveira 2014
		Delomys dorsalis (Hensel, 1872)	Thomasomys dorsalis collinus, D. collinus	82	80	-	Atlantic Forest of Southeastern Brazil, from MG and ES to RS	Musser and Carleton 2005, Gonçalves and Oliveira 2014
		Delomys sublineatus (Thomas, 1903)	-	72	90	-	Atlantic Forest of Southeastern Brazil, from MG and ES to SC	Musser and Carleton 2005, Gonçalves and Oliveira 2014
		Juliomys ossitenuis L. P. Costa, Pavan, Leite, and Fagundes, 2007	-	20	36	-	Southern ES, and Eastern SP and MG	Costa et al. 2007, Bonvicino et al. 2008
		Juliomys pictipes (Osgood, 1933)	Wilfredomys pictipes	36	34	-	Southeastern Brazil, from MG to RS	Bonvicino and Otazu 1999, Musser and Carleton 2005
		Juliomys rimofrons J. A. Oliveira & Bonvicino, 2002	-	20	34	-	High altitudes at Serra da Mantiqueira, in SP, RJ and MG	Oliveira and Bonvicino 2002, Bonvicino et al. 2008
		Juliomys sp.	-	32	48	-	Aparados da Serra National Park, ES	Paresque et al. 2009
		Phaenomys ferrugineus (Thomas, 1917)	-	78	114	-	Restricted areas from Serra do Mar, in RJ and SP	Bonvicino et al. 2001b, Musser and Carleton 2005
		Wilfredomys oenax (Thomas, 1928)	-	N/A	N/A	-	Southern Brazil and Southeastern SP	Bonvicino et al. 2008
**Family Ctenomyidae**		Ctenomys bicolor Miranda-Ribeiro, 1914	-	40	64	-	RO	Stolz 2012
		Ctenomys flamarioni Travi, 1981	-	48	50-78	Variation in the amount of constitutive heterochromatin	Eastern RS	Massarini and Freitas 2005, Bonvicino et al. 2008
		Ctenomys ibicuiensis T. R. O. Freitas, Fernandes, Fornel & Roratto, 2012	-	50	68	-	Western RS	Bidau 2015
		Ctenomys lami T. R. O. Freitas, 2001	-	54-58	74-82; 84	Centric fusion/ fission in pairs 1 and 2; pericentric inversion	RS (Coxilha das Lombas, Northeastern Guaiba River to Southwestern Banks of Barros Lake)	Woods and Kilpatrick 2005, Freitas 2007
		Ctenomys minutus Nehring, 1887	-	42, 43, 44; 45; 46-51; 49-51; 48-51; 51; 52	74; 75/76; 77; 78; 78, 80; 79	Robertsonian rearrengements and tandem fusions	Eastern RS and SC	Freitas 1997, Gava and Freitas 2002, Freygang et al. 2004, Bonvicino et al. 2008
		Ctenomys nattereri Wagner, 1848	Ctenomys boliviensis	36	64	-	Southwestern MT and Southeastern RO	Anderson et al. 1987, Bonvicino et al. 2008, Stolz 2012
		Ctenomys rondoni Miranda-Ribeira, 1914	-	N/A	N/A	-	MT and RO	Bidau 2015
**Family Ctenomyidae**		Ctenomys torquatus Lichtenstein, 1830	-	40, 42, 44, 46	72	Robertsonian fusion; Variation in the amount of constitutive heterochromatin; secondary constricton	Southeastern RS	Freitas and Lessa 1984, Bonvicino et al. 2008, Fernandes et al. 2009
**Family Cuniculidae**		Cuniculus paca (Linnaeus, 1766)	-	74	98	-	All Brazilian States	Giannoni et al. 1991, Bonvicino et al. 2008
**Family Dasyproctidae**		Dasyprocta azarae Lichtenstein, 1823	Dasyprocta aurea	64	122	-	Southcentral Brazil, MG and SP	Souza et al. 2007, Bonvicino et al. 2008
		Dasyprocta croconota Wagler, 1831	-	N/A	N/A	-	Northeastern PA, Northwestern CE and Northermost TO	Bonvicino et al. 2008, Patton and Emmons 2015
		Dasyprocta fuliginosa Wagler, 1832	-	64; 65	116; 122	B chromosome	AM, AC, RO and Northwestern MT	Lima and Langguth 1998, Ramos et al. 2003, Bonvicino et al. 2008
		Dasyprocta iacki Feijó & Langguth, 2013	Dasyprocta aguti	64	122	-	Littoral zone in PB and PE	Lima and Langguth 1998, Feijó and Langguth 2013, Patton and Emmons 2015
		Dasyprocta leporina Linnaeus, 1758	-	64, 65	122-124	B chromosome	Northermost Brazil (AM, RR, AP and PA)	Ramos et al. 2003, Bonvicino et al. 2008, Patton and Emmons 2015
		Dasyprocta prymnolopha Wagler, 1831	Dasyprocta nigriclunis	64, 65	122	B chromosome	Northeastern Brazil, and Northern MG	Ramos et al. 2003, Woods and Kilpatrick 2005, Bonvicino et al. 2008
		Dasyprocta punctata Gray, 1842	-	N/A	N/A	-	Southeastern Brazil	Woods and Kilpatrick 2005, Patton and Emmons 2015
		Dasyprocta variegata Tschudi, 1845	-	64*	124	-	Western Brazil	Patton and Emmons 2015
		Dasyprocta sp.	-	64, 65	124	B chromosome	unknown distribution	Ramos et al. 2003
		Myoprocta acouchy (Erxleben, 1777)	-	62	118	-	RR, and Northeastern AM and PA	Hsu and Benirschke 1968, Bonvicino et al. 2008, Patton and Emmons 2015
		Myoprocta pratti Pocock, 1913	-	N/A	N/A	-	AC and Western AM	Bonvicino et al. 2008, Patton and Emmons 2015
**Family Dinomyidae**		Dinomys branickii Peters, 1873	-	64	98	-	AC and Southwesternmost AM	Bonvicino et al. 2008, Vargas and Ortiz 2010
**Family Echimyidae**		Callistomys pictus (Pictet, 1843)	-	42	76	-	Southeastern BA	Bonvicino et al. 2008, Ventura et al. 2008, Emmons and Leite 2015
		Carterodon sulcidens (Lund, 1838)	-	66	N/A	Secondary constriction in the forth largest pair	DF, GO, MT and MG	Carmignotto 2005, Bezerra and Bonvicino 2015, Present study
**Family Echimyidae**		Clyomys laticeps (Thomas, 1909)	Clyomys bishopi	34; 32	58, 60, 62; 54	Pericentric inversion; Robertsonian rearrangement; secondary constriction in pair 1; addition of constitutive heterochromatin	MT, MS, GO, DF, SP and MG	Souza and Yonenaga-Yassuda 1984, Svartman 1989, Bonvicino et al. 2008, Bezerra et al. 2012
		Dactylomys boliviensis Anthony, 1920	-	118	168	-	AC	Dunnum et al. 2001, Woods and Kilpatrick 2005
		Dactylomys dactylinus (Desmarest, 1817)	-	94	144	-	AM, PA, RR, TO and Northern GO	Aniskin 1993, Bonvicino et al. 2008
		Echimys chrysurus (Zimmermann, 1780)	-	N/A	N/A	-	Southern AP, Northeastern PA and Northwestern MA	Bonvicino et al. 2008
		Echimys vieirai Iack-Ximenes, de Vivo & Percequillo, 2005	-	N/A	N/A	-	Central-Easternmost AM and Central-Westernmost PA	Bonvicino et al. 2008
		Euryzygomatomys spinosus (G. Fischer, 1814)	-	46	82	-	Eastern MG, SP and RJ, PR and Northern RS	Yonenaga 1975, Bonvicino and Bezerra 2015
		Isothrix bistriata Wagner, 1845	-	60	116	-	Northern AC and RO, Northeastern MT, and Southern AM	Leal-Mesquita 1991, Bonvicino et al. 2008
		Isothrix negrensis Thomas, 1920	-	60	112	-	Northern AM	Bonvicino et al. 2003b, Bonvicino et al. 2008
		Isothrix pagurus Wagner, 1845	-	22	38	-	Northeastern AM	Patton and Emmons 1985, Bonvicino et al. 2008
		Kannabateomys amblyonyx (Wagner, 1845)	-	98	126	-	Eastern Brazil, from ES to RS	Paresque et al. 2004, Bonvicino et al. 2008
		Lonchothrix emiliae Thomas, 1920	-	N/A	N/A	-	Eastern AM	Bonvicino et al. 2008
		Makalata didelphoides (Desmarest, 1817)	-	66	106	Secondary constriction in pair 11	AP, RR, Eastern AM, Western PA and TO, and Northern MT	Lima et al. 1998, Bonvicino et al. 2008
		Makalata macrura (Wagner, 1842)	-	N/A	N/A	-	AM and AC	Bonvicino et al. 2008
		Makalata obscura (Wagner, 1840)	-	N/A	N/A	-	Eastern PA and Westernmost MA	Bonvicino et al. 2008
		Mesomys hispidus (Desmarest, 1817)	-	60	116	-	Northern Brazil, and Northwestern MT	Leal-Mesquita 1991, Bonvicino et al. 2008
		Mesomys occultus Patton, da Silva & Malcolm, 2000	-	42	54	Secondary constriction in the smallest biarmed pair	Central AM	Patton et al. 2000, Woods and Kilpatrick 2005
		Mesomys stimulax Thomas, 1911	-	60	116	-	Eastern PA	Patton et al. 2000, Bonvicino et al. 2008
		Myocastor coypus (G. I. Molina, 1782)	-	42	76	-	RS	González and Brum-Zorilla 1995, Bonvicino et al. 2008, Fabre et al. 2016
**Family Echimyidae**		Phyllomys blainvillii (Jourdan, 1837)	-	50	88, 94-96	Pericentric inversion	BA, SE, AL and PE, Southern CE, and Northern MG	Souza 1981, Leite 2003, Bonvicino et al. 2008, Machado 2010
		Phyllomys brasiliensis Lund, 1840	-	N/A	N/A	-	Central MG	Bonvicino et al. 2008
		Phyllomys dasythrix Hensel, 1872	-	72	108	-	Southern PR to RS	Leite 2003, Woods and Kilpatrick 2005, Machado 2010
		Phyllomys kerri (Moojen, 1950)	-	N/A	N/A	-	Ubatuba (SP)	Woods and Kilpatrick 2005
		Phyllomys lamarum (Thomas, 1916)	-	56	102	-	Eastern Brazil, from PB to MG	Woods and Kilpatrick 2005, Araújo et al. 2014
		Phyllomys lundi Y. L. R. Leite, 2003	-	N/A	N/A	-	Southern MG to RJ	Bonvicino et al. 2008
		Phyllomys mantiqueirensis Y. L. R. Leite, 2003	-	N/A	N/A	-	Serra da Mantiqueira (MG)	Bonvicino et al. 2008
		Phyllomys medius (Thomas, 1909)	-	96	108	-	From RJ to RS	Sbalqueiro et al. 1989, Bonvicino et al. 2008
		Phyllomys nigrispinus (Wagner, 1842)	-	84, 85	N/A	Secondary constriction in one acrocentric pair	Coast from RJ to PR, extending to inland Western SP	Leite 2003, Woods and Kilpatrick 2005, Delciellos et al. 2017
		Phyllomys pattoni Emmons, Leite, Kock & Costa, 2002	-	72; 76; 80	114; 148; 100, 108, 112	Pericentric inversion; centric fusion/ fission	From PB to Northeastern SP	Zanchin 1988, Leite 2003, Paresque et al. 2004, Woods and Kilpatrick 2005, Leite and Loss 2015
		Phyllomys sulinus Y. L. R. Leite, Christoff & Fagundes, 2008	-	92	102	-	Southern Brazil, from SP to RS	Yonenaga 1975, Leite 2003, Leite and Loss 2015
		Phyllomys thomasi (Ihering, 1897)	-	N/A	N/A	-	Ilha de São Sebastião (SP)	Woods and Kilpatrick 2005, Leite and Loss 2015
		Phyllomys unicolor (Wagner, 1842)	-	N/A	N/A	-	Southernmost BA	Bonvicino et al. 2008, Leite and Loss 2015
		Proechimys brevicauda (Günther, 1876)	-	28	48-50	Variations in FN due to difficulty in classifying the morphology of the small pairs	AC and Southern AM	Patton et al. 2000, Bonvicino et al. 2008
		Proechimys cuvieri Petter, 1978	-	28	46-48	Differences in the number of subtelocentrics and acrocentrics	Northern Brazil	Maia and Langguth 1993, Patton et al. 2000, Bonvicino et al. 2008
		Proechimys echinotrix M. N. F. da Silva, 1998	-	32	60	-	Northwestern AM	da Silva 1998, Bonvicino et al. 2008
		Proechimys gardneri M. N. F. da Silva, 1998	-	40	54, 56	Pericentric inversion; secondary constriction in the smallest submetacentric pair	Southern AM	da Silva 1998, Bonvicino et al. 2008, Eler et al. 2012
		Proechimys goeldii Thomas, 1905	-	24	44	-	Easternmost AM and Northwestern PA	Machado et al. 2005, Patton and Leite 2015
		Proechimys gr. goeldii	-	15	16	-	MT	Machado et al. 2005
**Family Echimyidae**		Proechimys guyannensis (I. Geoffrey St.-Hilaire, 1803)	-	38, 44	52	One name with different karyotypes associated	Northeastern AM, Northern PA, Southeastern RR and AP	Machado et al. 2005, Bonvicino et al. 2008
		Proechimys hoplomyoides Tate, 1939	-	N/A	N/A	-	Northernmost RR	Bonvicino et al. 2008
		Proechimys kulinae M. N. F. da Silva, 1998	-	34	52	-	Southeastern AM	da Silva 1998, Patton et al. 2000, Bonvicino et al. 2008
		Proechimys longicaudatus (Rengger, 1830)	-	28	48-50	Pericentric inversion of pairs 3 and 11; addition/deletion of constitutive heterochromatin	MT	Machado et al. 2005, Bonvicino et al. 2008
		Proechimys cf. longicaudatus	-	16, 17	14	Robertsonian rearrangement between X and the largest acrocentric chromosome; Multiple sex chromosome system (XX, XY1Y2)	MT	Amaral et al. 2013
		Proechimys pattoni M. N. F. da Silva, 1998	-	40	56	-	Western AC	Patton and Gardner 1972, da Silva 1998, Bonvicino et al. 2008
		Proechimys quadruplicatus Hershkovitz, 1948	-	28	42	-	Northcentral AM	Patton et al. 2000, Bonvicino et al. 2005b, Bonvicino et al. 2008
		Proechimys roberti Thomas, 1901	-	30	54-56	Pericentric inversion of pairs 13 and 14	Eastern PA, TO and GO, and Western MG and MA	Svartman 1989, Leal-Mesquita 1991, Machado et al. 2005, Ribeiro 2006, Bonvicino et al. 2008
		Proechimys simonsi Thomas, 1900	Proechimys hendeei	32	56-58	Pericentric inversion; secondary constriction in pair 8 of the karyotype with NF=56	AC and Southwestern AM	Patton and Gardner 1972, Gardner and Emmons 1984, Patton et al. 2000, Bonvicino et al. 2008
		Proechimys steerei Goldman, 1911	-	24	40-42	Pericentric inversion in pair 3 (smallest metacentric), with homo or heterozigous chromosomes	AC and Southwestern AM	Patton et al. 2000, Bonvicino et al. 2008
		Proechimys sp.	Proechimys gr. longicaudatus	30	52	-	Rio Jamari, RO	Leal-Mesquita 1991, Patton and Leite 2015
		Proechimys sp. A	Proechimys gr. goeldii	38	52	-	Rio Negro-Rio Aracá, AM	Bonvicino et al. 2005b
		Proechimys sp. B	-	46	50	-	RR and Northern AM	Bonvicino et al. 2005b, Bonvicino et al. 2008
		Thrichomys apereoides (Lund, 1839)	-	28	50, 52	Secondary constriction in pair 2	MG, Eastern GO and Western BA	Bonvicino et al. 2002a, Pessôa et al. 2004
		Thrichomys inermis (Pictet, 1843)	-	26	48	Secondary constriction in pair 2	BA and TO	Pessôa et al. 2004, Bonvicino et al. 2008
		Thrichomys laurentius Thomas, 1904	-	30	54	Secondary constriction in pair 1	Northeastern Brazil, except MA	Souza and Yonenaga-Yassuda 1982, Bonvicino et al. 2008
**Family Echimyidae**		Thrichomys aff. laurentius	-	30	56	Secondary constriction in pair 1	Central Brazil	Bonvicino et al. 2002a, Braggio and Bonvicino 2004
		Thrichomys pachyurus Wagner, 1845	-	34	64	Secondary constriction in pair 2	Southern MT, and MS	Pessôa et al. 2004, Bonvicino et al. 2008
		Trinomys albispinus (I. Geoffrey St.-Hilaire, 1838)	-	60	116	Secondary constriction in pair 10	BA, SE and MG	Leal-Mesquita et al. 1993, Souza et al. 2006, Pessôa et al. 2015
		Trinomys dimidiatus (Günther, 1876)	-	60	116	Secondary constriction in pair 10	RJ and Northern SP	Pessôa et al. 2004, Bonvicino et al. 2008
		Trinomys eliasi (Pessôa & Reis, 1993)	-	38	112	Secondary constriction in pair 10	RJ	Pessôa et al. 2005, Bonvicino et al. 2008
		Trinomys gratiosus (Moojen, 1948)	Trinomys gr. bonafidei	56	108	Secondary constriction in pair 10	Southcentral ES to Southwestern RJ	Zanchin 1988, Woods and Kilpatrick 2005
		Trinomys iheringi (Thomas, 1911)	Proechimys iheringi iheringi	60-65	116	1 to 5 B chromosomes; secondary constriction in pair 7	Coast from Southern RJ to Northern PR	Yonenaga-Yassuda et al. 1985, Fagundes et al. 2004, Bonvicino et al. 2008
		Trinomys mirapitanga Lara, Patton and Hingst- Zaher, 2002	-	N/A	N/A	-	BA	Lara et al. 2002, Woods and Kilpatrick 2005
		Trinomys moojeni (Pessôa, Oliveira & Reis, 1992)	-	56	106	-	Only known from the type locality (MG)	Corrêa et al. 2005, Woods and Kilpatrick 2005
		Trinomys paratus (Moojen, 1948)	-	58	112	Secondary constriction in long arm of a median size autosome	South-central ES and easternmost MG	Bonvicino et al. 2008, Lazar et al. 2017
		Trinomys setosus (Desmarest, 1817)	Trinomys s. setosus and Trinomys s. elegans	56	108, 104	NFs refer to each subspecies, respectively	Eastern Brazil, from SE to *ES* and MG	Bonvicino et al. 2008, Pêssoa et al. 2015
		Trinomys yonenagae (P. L. B. Rocha, 1996)	-	54	104	Secondary constriction in pair 10	BA, left bank of Rio São Francisco	Leal-Mesquita et al. 1992, Bonvicino et al. 2008
		Toromys grandis (Wagner, 1845)	-	N/A	N/A	-	Eastern AM and PA	Bonvicino et al. 2008
**Family Erethizontidae**		Chaetomys subspinosus Olfers, 1818	-	52	76	-	ES and Southeastern BA	Bonvicino et al. 2008, Vilela et al. 2009
		Coendou insidiosus (Olfers, 1818)	Sphiggurus insidiosus	62	76	-	Eastern Brazil, from CE to ES	Lima 1994, Bonvicino et al. 2008, Voss 2015c
		Coendou melanurus (Wagner, 1842)	Sphiggurus melanurus	72	76	-	Northernmost Brazil (AM, RR, AP and PA)	Bonvicino et al. 2002b, Bonvicino et al. 2008, Voss 2015c
		Coendou nycthemera (Olfers, 1818)	-	N/A	N/A	-	Easternmost AM and PA	Bonvicino et al. 2008, Voss 2015c
		Coendou prehensilis (Linnaeus, 1758)	-	74	82	-	From Northern to Southeastern Brazil	Lima 1994, Bonvicino et al. 2008, Voss 2015c
		Coendou roosmalenorum Voss and da Silva, 2001	Sphiggurus roosmalenorum	N/A	N/A	-	Centraleastern AM	Bonvicino et al. 2008, Voss 2015c
**Family Echimyidae**		Coendou speratus Mendes Pontes, Gadelha, Melo, de Sá, Loss, Caldara Junior, Costa & Leite, 2013	-	N/A	N/A	-	Eastern PE and AL	Mendes-Pontes et al. 2013, Voss 2015c
		Coendou spinosus (F. Cuvier 1823)	Sphiggurus spinosus, S. villosus	42	76	-	Southern Brazil, Southeastern MG, and Eastern SP and RJ	Mendes-Pontes et al. 2013, Voss 2015c
**Family Muridae**		Mus musculus Linnaeus, 1758	-	40	38	-	All Brazilian States	Bonvicino et al. 2008, present study
		Rattus rattus Linnaeus, 1758	-	38	58-59	Pericentric inversion in pair 8	All Brazilian States	Kasahara and Yonenaga-Yassuda 1981, Kasahara and Yonenaga-Yassuda 1984, Bonvicino et al. 2008
		Rattus norvegicus Berkenhout, 1769	-	42	64	-	All Brazilian States	Bianchi et al. 1969, Bonvicino et al. 2008
**Family Sciuridae**		Guerlinguetus aestuans (Linnaeus, 1766)	Guerlinguetus gilvigularis, G. poaiae	N/A	N/A	-	RR, AP, AM, PA and Central MT	Bonvicino et al. 2008, De Vivo and Carmignotto 2015
		Guerlinguetus brasiliensis (Gmelin, 1788)	Guerlinguetus alphonsei, G. henseli, G. ingrami	40	74, 76	Pericentric inversions	Disjunct distribution of Amazonian, Caatinga, and Coastal Brazil	Lima and Langguth 2002, Fagundes et al. 2003, De Vivo and Carmignotto 2015
		Hadrosciurus igniventris (Wagner, 1842)	Sciurus igniventris	N/A	N/A	-	Northern Brazil, Southern Amazon River	Bonvicino et al. 2008, De Vivo and Carmignotto 2015
		Hadrosciurus pyrrhinus (Thomas, 1898)	Sciurus igniventris, S. pyrrhonotus, S. pyrrhinus	N/A	N/A	-	Western Brazilian Amazonia	Patton et al. 2015
		Hadrosciurus spadiceus (Olfers, 1818)	Sciurus spadiceus	40	76	-	Central to Southern AM, AC, RO, and Western PA and MT	Lima and Langguth 2002, Bonvicino et al. 2008, De Vivo and Carmignotto 2015
		Microsciurus flaviventer (Gray, 1867)	-	N/A	N/A	-	Northern Amazon River, Brazil	Bonvicino et al. 2008
		Notosciurus pucheranii (Fitzinger, 1867)	Guerlinguetus ignitus	N/A	N/A	-	Northwestern MT, Western AC and Southwestern AM	Bonvicino et al. 2008
		Sciurillus pusillus (I. Geoffrey St.-Hilaire, 1803)	-	N/A	N/A	-	Eastern AM and Western PA	Bonvicino et al. 2008

Abbreviations: Brazilian states AC: Acre; AL: Alagoas; AP: Amapá; AM: Amazonas; BA: Bahia; CE: Ceará; DF: Distrito Federal; ES: Espírito Santo; GO: Goiás; MA: Maranhão; MG: Minas Gerais; MS: Mato Grosso do Sul; MT: Mato Grosso; PA: Pará; PB: Paraíba; PE: Pernambuco; PI: Piauí; PR: Paraná; RJ: Rio de Janeiro; RN: Rio Grande do Norte; RO: Rondônia; RR: Roraima; RS: Rio Grande do Sul; SC: Santa Catarina; SE: Sergipe; SP: São Paulo; TO: Tocantins. N/A means that information is not available and (*) means that data do not refer to Brazilian specimens.

### Sampling

The single female of Carterodon sulcidens (lab number: CIT787/ field number: APC58) was captured in Serra da Mesa, State of Goiás, Brazil (13°53'S, 48°19'W), a region characterized by the Cerrado biome. Additionally, five males of Mus musculus (field number: PCH4078, 4079, 4094–96) were captured in Guará, São Paulo State, Brazil (20°29'S, 47°51'W), a transitional region between the Cerrado and Atlantic Forest.

Regarding Neacomys, four specimens of N. amoenus amoenus Thomas, 1903 were captured in Mato Grosso State, Brazil, in a transitional area between Cerrado and Amazonian Rainforest. Two specimens of Neacomys sp. were captured, one at Vila Rica (Mato Grosso State), and the other at Igarapé-Açu (Amazonas State), Brazil (field number, locality, and coordinates are presented in Suppl. material 1).

### Cytogenetic preparation

Chromosome preparations of Carterodon sulcidens, the five samples of Mus musculus, four Neacomys a. amoenus, and a specimen of Neacomys from Vila Rica, Mato Grosso State, were obtained *in vivo* from bone marrow and spleen, following Ford and Hamerton (1956) or *in vitro* from fibroblast culture (Freshney 1986). Conventional Giemsa staining was performed to determine the diploid and fundamental numbers, and C-banding and Ag-NOR were performed according to Sumner (1972) and Howell and Black (1980), respectively.

### Molecular phylogeny analyses of Neacomys

DNA was extracted from the liver or muscle with Chelex 5% (Bio-Rad) (Walsh et al. 1991) of five specimens of Neacomys. DNA of the specimen from Vila Rica, Mato Grosso State, was extracted from fibroblast cell culture using DNeasy Blood and Tissue kit (Qiagen, catalog number 69506).

PCR was performed in a thermal cycler (Eppendorf Mastercycler ep Gradient, Model 5341) using primers MVZ05 (5-CGA AGC TTG ATA TGA AAA ACC ATC GTT G-3) and MVZ16 (5-AAA TAG GAA RTA TCA YTC TGG TTT RAT-3) (Irwin et al. 1991, Smith and Patton 1993, respectively). PCR mixture contained 30 ng of DNA, 25 pmol of each primer, 0.2 mM of dNTP, 2.52 µL of reaction buffer (50 mM KCl, 2.5 mM MgCl_2_, 10 mM Tris-HCl; pH 8.8) and 0.2 units of Taq DNA polymerase (Invitrogen). Thirty-nine amplification cycles were performed, consisting of denaturation at 94 °C for 30 s, annealing at 48 °C for 45 s, extension at 72 °C for 45 s and the final extension at 72 °C for 5 min. The PCR products were separated using 1% agarose gel in TAE buffer. Sequencing was conducted using BigDye (DNA “Big Dye Terminator Cycle Sequencing Standart,” Applied Biosystems) and an ABI PRISM 3100 Genetic Analyzer (Applied Biosystems). All sequences were submitted to a comparative similarity search on BLAST (Basic Local Alignment Search Tool) before the alignment. Alignments were performed by using Muscle (Edgar, 2004) implemented in Geneious 4.8.5 (Biomatters). GenBank access numbers are provided in Suppl. material 1.

Models of nucleotide substitution were selected using Bayesian Information Criterion (BIC), implemented in PartitionFinder, version 1.1.1 (Lanfear et al. 2012). Approximately 673 bp were used to perform Maximum Likelihood (ML) in GARLI 2.0 (Bazinet et al. 2014) and Bayesian Inference (BI) in MrBayes 3.04b (Ronquist and Huelsenbeck 2003), using 69 additional Neacomys sequences downloaded from GenBank, plus sequences of Euryoryzomys russatus (Wagner, 1848), Holochilus brasiliensis (Desmarest, 1819) and Oligoryzomys nigripes (Olfers, 1818) as the outgroup (see Suppl. material 1).

## Results

The current review encompasses all rodent species which up to the present have been reported in Brazil, comprising 271 species from 10 families (Musser and Carleton 2005, Patton et al. 2015, Fabre et al. 2016). Diploid number ranges from 2n = 9, 10 in Akodon sp. n. to 2n = 118 in Dactylomys boliviensis Anthony, 1920 (Table 1). It is noteworthy that 38 species (14%) lack any cytogenetic data. Besides, nine species present only the diploid number with no information about the fundamental number.

Many species show chromosome rearrangements leading to variation in diploid and fundamental numbers. Also, more than one diploid number was associated with one single species, suggesting that they could represent species’ complexes. Additionally, new karyotypes were assigned to 22 species highlighting them as candidate species, which have not been formally described yet.

All comments below refer to the data compiled and presented in Table 1.

### Family Caviidae

From a total of ten species, cytogenetic data is lacking for only one: Galea flavidens (Brandt, 1835). The diploid number varied from 2n = 52 in Kerodon acrobata Moojen, 1997 and K. rupestris (Wied-Neuwied, 1820) to 2n = 66 in Hydrochoerus hydrochaeris (Linnaeus, 1766). Currently, polymorphism of autosomal chromosomes has been described for Cavia porcellus (Linnaeus, 1758), pericentric inversions for C. magna Ximénez, 1980 and K. rupestris, and Robertsonian rearrangement for C. magna (Maia 1984, Gava et al. 2011) (Table 1).

### Family Cricetidae

#### Subfamily Sigmodontinae

##### Tribe Akodontini

This is the second most diverse tribe in the subfamily Sigmodontinae. Only five out of 42 species (D’Elía and Pardiñas 2015) that occur in Brazil lack diploid number information (Table 1). However, for one species, Akodon toba Thomas, 1921, such information is available only for Paraguayan specimens. In addition to the species on which there is no information on the diploid number, four species of the genus Oxymycterus Waterhouse, 1837 have not had their fundamental number established, yet.

In this tribe, the diploid number varied from 2n = 9, 10 in Akodon sp. n. to 2n = 70 in Bibimys labiosus (Winge, 1887). B chromosomes are found in Akodon montensis and Blarinomys breviceps (Winge, 1887). Also, pericentric inversions were described in three species of the tribe, Robertsonian rearrangements in six, and reciprocal translocation in one. These rearrangements are reported for Akodon cursor (although some authors consider A. cursor as a species complex, because of the molecular phylogeny – see Geise et al. 2001, Silva et al. 2006), Akodon sp. n., Akodon montensis, Blarinomys breviceps, Brucepattersonius griserufescens Hershkovitz, 1998, Deltamys kempi Thomas, 1917, Necromys lasiurus (Lund, 1840), Scapteromys meridionalis Quintela, Gonçalves, Althoff, Sbalqueiro, Oliveira, & Freitas, 2014, and Thalpomys lasiotis Thomas, 1916.

Sex chromosome variation is also common, occurring in six species. It is also remarkable that Deltamys kempi is one of the few rodents to which multiple sex system has been described (X_1_X_1_X_2_X_2_/ X_1_X_2_Y) (Sbalqueiro et al. 1984).

Cytogenetic studies have proved to be a useful tool in the recognition of species, mainly in the case of the cryptic and sympatric species as Akodon cursor and A. montensis. On the other hand, karyotype was less variable in some other Akodontini genus (for instance Brucepattersonius and Oxymycterus), and in this case, they could not be distinguished cytogenetically. This reveals the need for gathering cytogenetic, molecular and morphological data in taxonomic studies.

##### Tribe Ichthyomyini

Two species of Neusticomys, N. oyapocki (Dubost & Petter, 1979) and N. ferreirai Percequillo, Carmignotto & Silva, 2005, occur in Brazil and karyotype information is available only for N. ferreirai (Table 1). Karyotype shows 2n = 92, FN = 98, and autosomes consist of four biarmed pairs and 41 acrocentrics. X chromosome is a large metacentric and Y is the largest acrocentric (Percequillo et al. 2005).

##### Tribe Oryzomyini

Comprising 73 species up to now, this tribe alone comprises about 47% of the Sigmodontinae diversity. Notwithstanding, it is one of the best cytogenetically studied taxa of Brazilian rodents, and cytogenetic information on fundamental number lacks for only one species: Neacomys guianae Thomas, 1905. In Brazilian representatives the diploid number varied from 2n = 34 in Neacomys musseri Patton, da Silva & Malcolm, 2000 to 2n = 86 in Zygodontomys brevicauda (J. A. Allen & Chapman, 1893).

Pericentric inversion (n = 13) and Robertsonian rearrangements (n = 8) are common rearrangements, as well as sex chromosomes variations, that were described in 12 species and correlated to addition/deletion of constitutive heterochromatin and pericentric inversions.

Besides, Oryzomyini is also the tribe with more species having supernumerary chromosomes (n = 6). Remarkably, B chromosomes in this tribe present different morphology and composition, not only between, but also within the same species. For instance, Nectomys squamipes Brants, 1827 presents from one to three supernumeraries that could be large/medium submetacentric or medium acrocentric, with interstitial or entire long arm C-banded, with late or early replication and with or without interstitial telomeric sites (Silva and Yonenaga-Yassuda 1998b). Differences were also described in Bs of Holochilus brasiliensis, Nectomys rattus Pelzeln, 1883, and Oligoryzomys flavescens (Waterhouse, 1837) (Silva and Yonenaga-Yassuda 2004). Recently, FISH with Holochilus brasiliensis probes of sex chromosomes (X and Y) and both supernumeraries (B1 and B2) were performed, revealing positive signal on sex chromosome of 12 Oryzomyini species and Bs of Holochilus brasiliensis, Nectomys rattus and N. squamipes (Ventura et al. 2015). No signal was observed in Bs of Oligoryzomys flavescens and Sooretamys angouya (G. Fischer, 1814), though, corroborating that supernumeraries in this group may have had independent origins (Ventura et al. 2015).

Karyotype information proved to be important in this tribe, since many species present species-specific karyotypes. For example, species of the genus Oligoryzomys are morphologically very similar but they present different karyotypes: Oryzomys mattogrossae (J. A. Allen, 1916) (2n = 62, FN = 64), Oryzomys microtis (J. A. Allen, 1916) (2n = 64, FN = 64,66), Oryzomys moojeni Weksler & Bonvicino, 2005 (2n = 70, FN = 72, 74, 76), Oryzomys nigripes (2n = 62, FN = 80-82), Oryzomys stramineus Bonvicino & Weksler, 1998 (2n = 52, FN = 68-70), Oryzomys utiaritensis J. A. Allen, 1916 (2n = 72, FN = 76) (Almeida and Yonenaga-Yassuda 1991, Bonvicino and Weksler 1998, Andrades-Miranda et al. 2001a, Agrellos et al. 2012, Di-Nizo 2013).

Chromosome data also show evidence that distinctive karyotypes are being attributed to the same name, for instance Euryoryzomys macconnelli (Thomas, 1910), E. lamia (Thomas, 1901), Hylaeamys yunganus (Thomas, 1902), Oecomys cleberi Locks, 1981, Oecomys paricola (Thomas, 1904), Oecomys roberti (Thomas, 1904) and Zygodontomys brevicauda (Andrades-Miranda et al. 2000, Patton et al. 2000, Suárez-Villota et al. 2017).

Additionally, some species could not be identified by chromosome data alone, because they share the same karyotype. This is the case of Cerradomys marinhus (Bonvicino, 2003) and Pseudoryzomys simplex (Winge, 1887) (2n = 56, FN = 54 - except for the morphology of the Y); Euryoryzomys emmonsae (Musser et al., 1998), E. russatus and E. nitidus (Thomas, 1884) (2n = 80, FN = 86); Hylaeamys laticeps (Lund, 1840) and H. seuanezi (Weksler et al., 1999) (2n = 48, FN = 60); H. oniscus (Thomas, 1904) and H. perenensis (J. A. Allen, 1901) (2n = 52, FN = 62); Neacomys dubosti Voss et al., 2001 and N. amoenus (2n = 64, FN = 68); Oecomys bahiensis (Hershkovitz, 1960), Oecomys catherinae, and Oecomys concolor (Wagner, 1845), Oecomys sp. 2 and sp. 3 (2n = 60, FN = 62); Drymoreomys albimaculatus Percequillo, Weksler & Costa, 2011 and Oecomys sp. 4 (2n = 62, FN = 62 - although ITS was observed in Drymoreomys but not in Oecomys – see Suárez-Villota et al. 2013 and Malcher et al. 2017); and Holochilus brasiliensis and Nectomys squamipes (standard karyotypes: 2n = 56, FN = 56). Also, although not distributed in Brazil, Oligoryzomys brendae Massoia, 1998 is found sympatric to Oryzomys chacoensis (Myers & Carleton, 1981) in Argentina and both possess 2n = 58, FN = 74.

Just as in all hierarchical levels of rodents’ taxonomy, cytogenetic diversity is underestimated in this tribe. For instance, recently, Silva et al. (2015) described two new cytotypes for Neacomys: 2n = 58, FN = 64, from samples collected in Marabá, and 2n = 58, FN = 70, from samples collected in Chaves, Marajó Island, localities from Pará State. According to the authors, both cytotypes differed in the number of biarmed pairs due to amplification/deletion of constitutive heterochromatin in the short arms of pairs 24, 26, and 27 (from Marajó Island) and pericentric inversion involving pairs 28 (metacentric) and 24 (acrocentric) from Marajó Island and Marabá, respectively. These karyotypes could not be assigned to any species described so far, and molecular phylogeny of these samples corroborates the cytogenetic data that it might be a new species (Silva et al. 2015).

**Figure 1.**
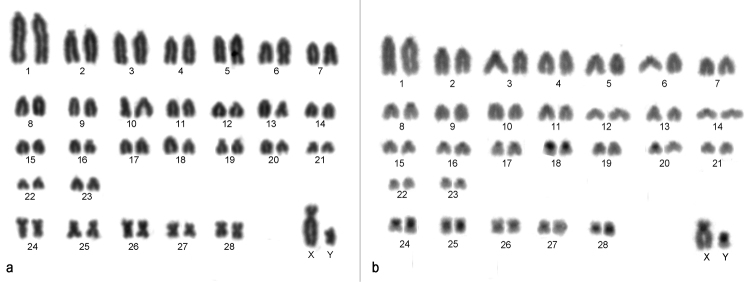
Karyotype of a male of Neacomys 2n=58, FN=66, from Vila Rica, Mato Grosso State, Brazil. **a** Giemsa-staining **b** C-banding.

Herein, we describe the same diploid (2n = 58), but with a different fundamental number (66) to Neacomys collected in Vila Rica, Mato Grosso State (approximately 700 km from those samples described by Silva et al. 2015). The karyotype comprises 23 acrocentric pairs decreasing in size (pair 1 is the largest of the complement), and five small biarmed pairs. The X chromosome is a large submetacentric, and the Y is a small submetacentric (Fig. 1a). The C-banding pattern shows constitutive heterochromatin at the pericentromeric regions of all autosomes, and in the short arm of both X and Y (Fig. 1b).

**Figure 2.**
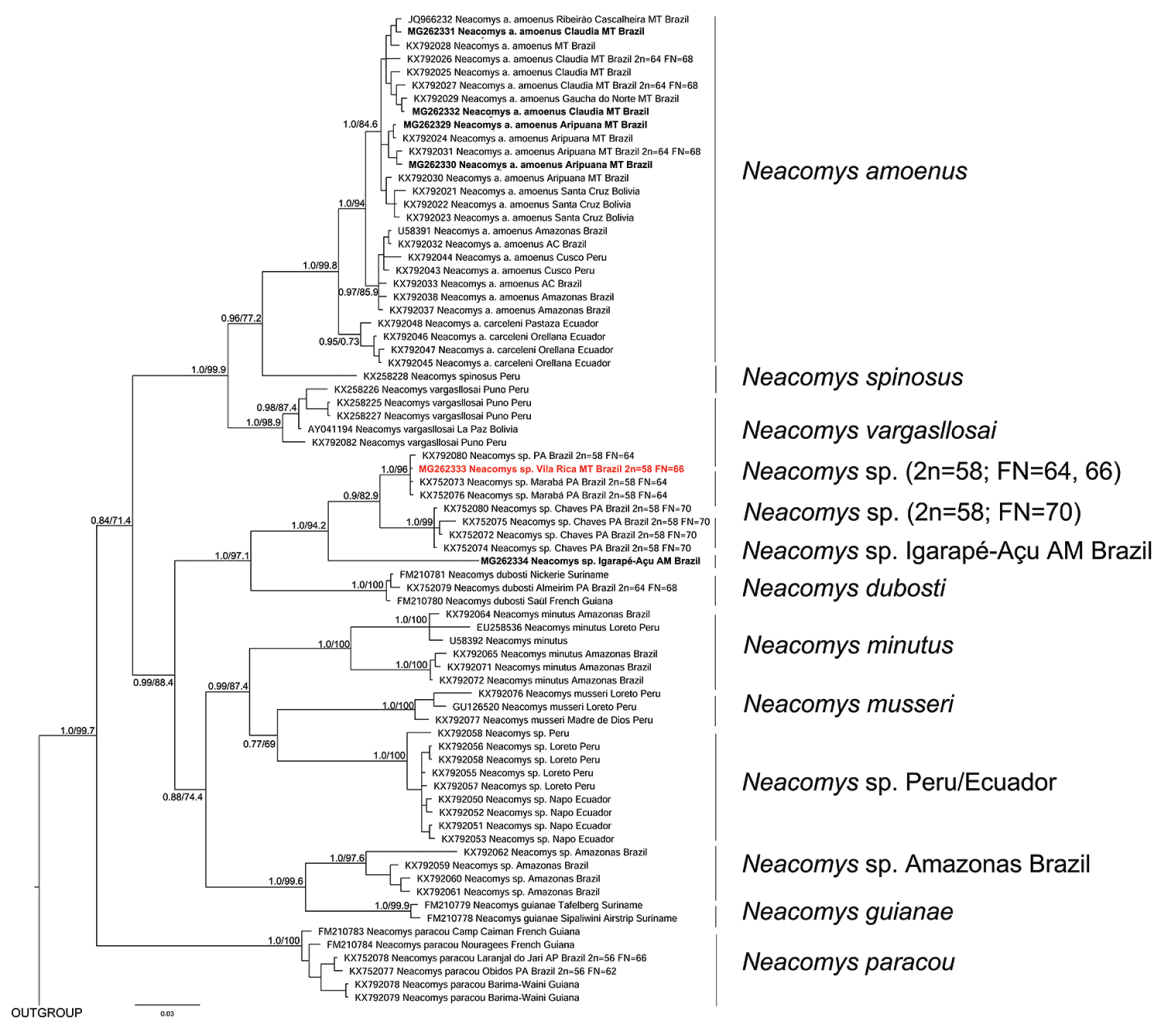
Bayesian phylogenetic hypothesis of Neacomys based on cyt-*b.* Numbers in the nodes indicate BI posterior probability (PP) and bootstrap support (ML), respectively. Individual from Vila Rica, Mato Grosso State with 2n=58, FN=66, is highlighted in red and the other samples analysed in this work are in bold.

For phylogenetic analyses, the best model selected for the mitochondrial gene (cyt-*b*) was GTR+I+G. Our molecular phylogeny suggests that this specimen with 2n = 58, FN = 66, from Vila Rica may be an undescribed species that belongs to the same one reported by Silva et al. (2015) with 2n = 58, FN = 64, but with a new fundamental number, probably due to pericentric inversions (Fig. 2). Two structured clades of Neacomys with 2n = 58 were recovered: one with samples with FN = 70, and the other with FN = 64 and 66. Additionally, a sample from Igarapé-Açu (MTR12842), Rio Abacaxis (Amazonas, Brazil) was recovered as the sister group of these two clades. Although the phylogenetic reconstruction lacks N. tenuipes Thomas, 1900 (because the unique sequence available in GenBank has only 177pb), it is unlikely that samples with 2n = 58 belong to N. tenuipes once this species is distributed in Colombia and Venezuela and did not nest in the clade of N. tenuipes of the molecular phylogeny presented by Silva et al. (2015). In addition, our phylogenetic reconstruction recovered Neacomys as monophyletic with high support values (1PP/ 99ML). ML and IB analyses recovered the same topology.

##### Tribe Phyllotini

In Brazil, this tribe was initially composed only of the genus Calomys Waterhouse, 1837. However, due to sampling efforts, a new genus was recently added, Calassomys Pardiñas, Lessa, Salazar-Bravo & Câmara, 2014. The diploid number varied from 2n = 36 in Calomys cerqueirai to 2n = 66 in Calomys tener and Calomys expulsus, although the latter presents two different diploid numbers and karyotypes associated to its name, therefore highlighting the need for further investigation (Bonvicino and Almeida 2000, Mattevi et al. 2005). Cytogenetic information is available for all the representatives, and it is an important tool for the recognition of species (cytotaxonomy). One species presents centric fusion (Calomys cerqueirai) (Colombi and Fagundes 2014).

##### Tribe Reithrodontini

In Brazil, the only representative of this tribe is Reithrodon typicus Waterhouse, 1837. This species possesses a low diploid number (2n = 28) and occurs on the border of Uruguay (Freitas et al. 1983, Pardiñas et al. 2015c) (Table 1).

##### Tribe Sigmodontini

Only one species of this tribe can be found in Brazil, Sigmodon alstoni (Thomas, 1881). Voss (1992) karyotyped 11 specimens from three localities at Venezuela with 2n = 78, 80 and 82, but the picture of the karyotypes and the fundamental numbers were not reported. Also, the author suggested that Robertsonian rearrangement is a plausible explanation for the variation observed. There have been no Brazilian representatives of this species karyotyped so far.

##### Tribe Thomasomyini

This tribe is represented by only two genera in Brazil: Rhipidomys Tschudi, 1845 and Rhagomys Thomas, 1886. The diploid number varied from 2n = 36 in Rhagomys rufescens (Thomas, 1886) to 2n = 50 in Rhipidomys nitela Thomas, 1901. Apart from R. nitela, which possesses 2n = 48 (samples from Roraima State) or 50 (samples from Manaus, Amazonia State), in general, the karyotype is not informative for Rhipidomys, since nine species present the same diploid number (2n = 44), and two species lack karyotype data (Silva and Yonenaga-Yassuda 1999, Tribe 2005). In fact, Tribe (2015) provisionally inserted the 2n = 50 samples in R. nitela but reiterated that they need taxonomic revision. Pericentric inversion, found in six species, plays an important role in the genus, and this is reflected in the variation of the fundamental number. Two species lack cytogenetic data: Rhipidomys ipukensis R. G. Rocha, Costa & Costa, 2011 and R. wetzeli A. L. Gardner, 1990.

##### Tribe Wiedomyini

This tribe is composed of two species: Wiedomys pyrrhorhinos (Wied- Neuwied, 1821) and W. cerradensis P. R. Gonçalves, Almeida & Bonvicino, 2005. Both occur in Brazil with disjunctive distribution (W. pyrrhorhinos at Caatinga, and W. cerradensis at Cerrado) and possess different karyotypes (2n = 62 and 60, respectively) (Maia and Langguth 1987, Gonçalves et al. 2005). Recent molecular studies indicate that W. pyrrhorhinos, may represent a species complex with Rio São Francisco acting as a barrier to the populations from both river banks (Di-Nizo *in prep*.). Pericentric inversions have also been described for this species.

### *Incertae sedis*

This group comprises the genera Abrawayaomys F. Cunha & Cruz, 1979, Delomys Thomas, 1917, Juliomys E. M. González, 2000, Phaenomys Thomas, 1917, and Wilfredomys Avila-Pires, 1960, which could not be inserted into any other tribes, according to phylogenetic and morphological analyses (Musser and Carleton 2005, Patton et al. 2015). Cytogenetic information is available for all species, except one, Wilfredomys oenax (Thomas, 1928), and is helpful for distinguishing species of the genus Delomys and Juliomys.

### Family Ctenomyidae

This family comprises a single genus, Ctenomys, which presents a great variation in diploid numbers, especially C. lami T. R. O. Freitas, 2001, C. minutus Nehring, 1887 and C. torquatus Lichtenstein, 1830 for which Robertsonian rearrangements and *in tandem* fusions were described (Freitas and Lessa 1984, Fernandes et al. 2009). The diploid number varied from 36 in Ctenomys nattereri Wagner, 1848 to 58 in C. lami. Only one species out of eight lacks karyotype information. Cytogenetic data was useful for recognizing Ctenomys bicolor Miranda- Ribeiro, 1914, C. ibicuiensis T. R. O. Freitas, Fernandes, Fornel & Roratto, 2012 and C. nattereri, because it presents exclusive karyotype (Stoulz 2012). Pericentric inversion has been described for C. lami and *in tandem* fusions for C. minutus.

### Family Cuniculidae

This family is represented by a single species, Cuniculus paca (Linnaeus, 1766), with a wide distribution and unique karyotype (2n = 74, FN = 98) (Giannoni et al. 1991, Bonvicino et al. 2008).

### Family Dasyproctidae

This family comprises two genera: Dasyprocta Illiger, 1811, with nine species, and Myoprocta Thomas, 1903, with two species (Patton and Emmons 2015). There is no cytogenetic data known for three species (Table 1). The diploid number in the Family varied from 62 to 65, and in the genus Dasyprocta, from 64 to 65, due to the presence of B chromosomes in four species (Ramos et al. 2003).

### Family Dinomyidae

This family possesses only one species, Dinomys branickii Peters, 1873, to which the karyotype is 2n = 64, FN = 98 (Table 1).

### Family Echimyidae

Even being the second largest Brazilian rodent family, a remarkable gap regarding cytogenetic data of this family still remains, with 14 species out of 68 lacking such information. This represents about 37% of all the unknown karyotypic information of all Brazilian rodents.

Diploid numbers varied from 2n = 15 in Proechimys goeldii Thomas, 1905 to 118 in Dactylomys boliviensis. B chromosomes have been described for one species: Trinomys iheringi (Thomas, 1911) (Yonenaga-Yassuda et al. 1985), pericentric inversion for seven species, and Robertsonian rearrangement for three. A multiple sex chromosome system was described for Proechimys cf. longicaudatus (Amaral et al. 2013), and addition/deletion of constitutive heterochromatin was described for Clyomys laticeps (Thomas, 1909) and P. longicaudatus (Rengger, 1830) (Souza and Yonenaga-Yassuda 1984, Bezerra et al. 2012, Machado et al. 2005). Secondary constriction is a characteristic feature of several species, occurring in Carterodon sulcidens (this work), Clyomys laticeps, Mesomys occultus Patton, da Silva & Malcolm, 2000, Makalata didelphoides (Desmarest, 1817), Proechimys gardneri M. N. F. da Silva, 1998, all five Thrichomys E.- L. Trouessart, 1880 species, and seven species of Trinomys Thomas, 1921.

Within this family, there are also cases in which different diploid numbers are assigned to the same name. In the case of Clyomys laticeps, the 2n = 34, FN = 58, 60, 62 and 2n = 32, FN = 54, the karyotypes are very similar, and differ by a Robertsonian rearrangement and pericentric inversion (2n = 32). Also, species such as Phyllomys pattoni Emmons, Leite, Kock & Costa, 2002 and Proechimys guyannensis E. Geoffroy, 1803 should be investigated by molecular phylogeny and morphology, because they are prone to either represent species-complex or have taxonomic misidentification.

**Figure 3.**
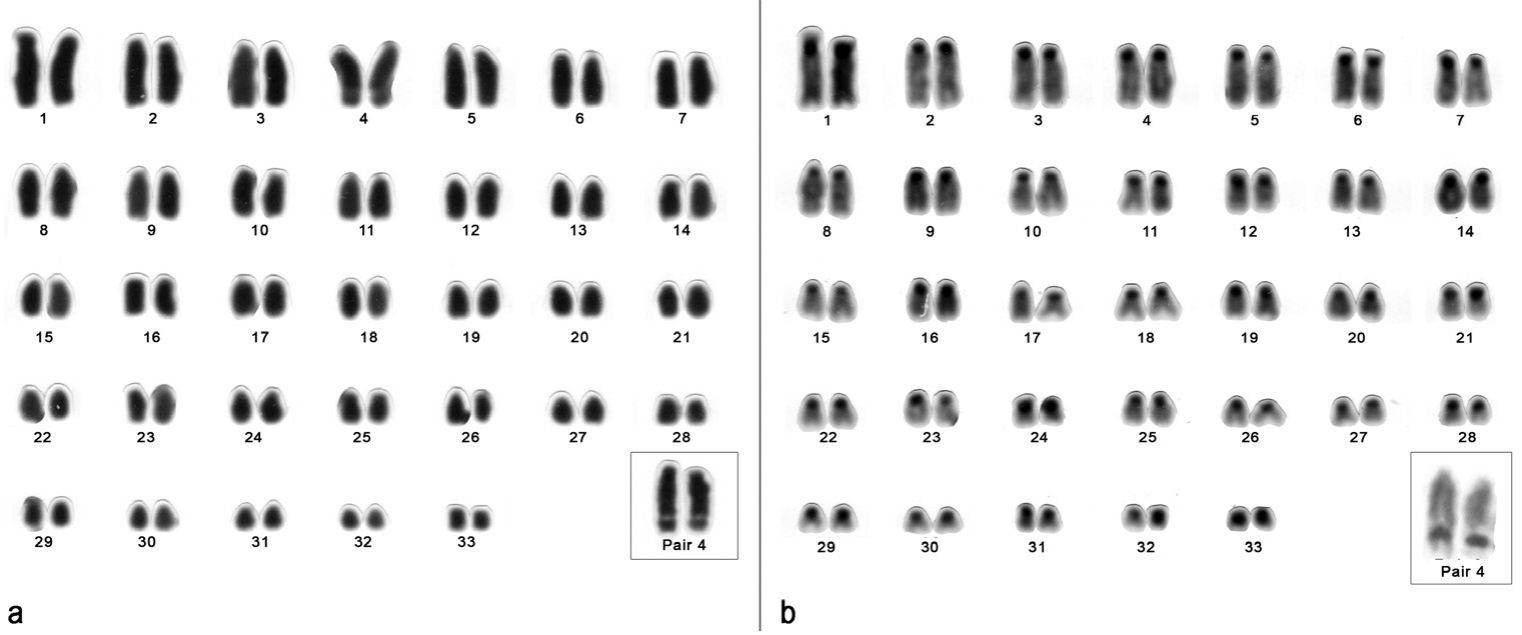
Karyotype of a female of Carterodon sulcidens with 2n=66 from Serra da Mesa, Goiás State, Brazil. **a** Giemsa-staining. Inset: Pair 4 with evident secondary constriction **b** C-banding. Inset: Pair 4 after silver nitrate staining.

In this work, the karyotype of Carterodon sulcidens is being described for the first time, showing 2n = 66. Since the animal was a female, it was not possible to recognize the X chromosomes and the exact morphology of the small pair, so we could not establish the fundamental number. Karyotype is composed of 32 acrocentric pairs decreasing in size and presumably one biarmed pair (pair 33). Also, the fourth largest pair possesses a remarkable secondary constriction (Fig. 3a). Constitutive heterochromatin is located in the pericentromeric region of all autosomes (Fig. 3b). Ag-NOR showed signals in the secondary constriction of pair 4 (Fig. 3b inset).

Within the Echimyidae Family, the only other species with 2n = 66 described so far is Makalata didelphoides, but its karyotype presents 20 pairs of metacentric chromosomes, which clearly differs from the karyotype of Carterodon sulcidens.

**Family Erethizontidae**

Three out of eight species lack cytogenetic information. The diploid number varied from 42 in Coendou spinosus (F. Cuvier, 1823) to 74 in C. prehensilis (Linnaeus, 1758) (Lima 1994, Mendes-Pontes et al. 2013) (Table 1).

### Family Muridae

This family (represented by the genera Mus and Rattus) was introduced from Europe, and even though it is not a native, it is currently widespread throughout Brazil (Musser and Carleton 2005).

**Figure 4.**
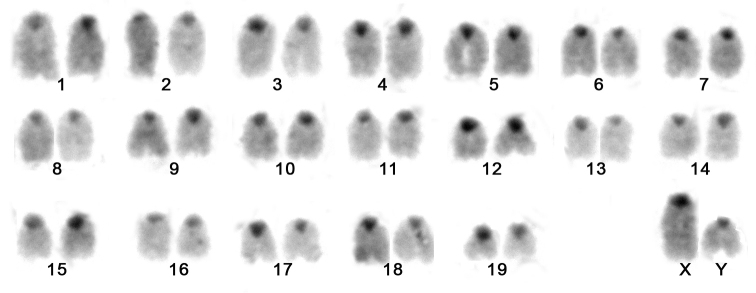
Karyotype after C-banding of a male of Mus musculus with 2n=40, FN=38, from Guará, São Paulo State, Brazil.

Little is known about the cytogenetics of the Mus musculus Brazilian populations because this species seems to be negglected. The present paper features the first picture of Mus musculus karyotype from Brazil. This species presented 2n = 40, FN = 38, with all chromosomes acrocentrics. C-banding was restricted to the centromeric region of all chromosomes (Fig. 4). Sex chromosomes could only be recognized after G-banding (not showed) because they have similar morphology compared to the autosomes.

For the black rat Rattus rattus Linnaeus, 1758, diploid number of South America population is the same as those from Oceania (2n = 38), and Kasahara and Yonenaga-Yassuda (1981) described pericentric inversion for individuals from São Paulo, Brazil.

### Family Sciuridae

Cytogenetic data is unknown for almost the entire family. For the two species to which chromosome information is known, diploid number is 2n = 40, and pericentric inversion has been described for one of them, Guerlinguetus brasiliensis (Gmelin, 1788) (Lima and Langguth 2002, Fagundes et al. 2003) (Table 1).

## Discussion

### Advances since the last revision

The last cytogenetic revision on Brazilian rodents, published in 1984, described the karyotype of 62 species, mainly from South and Southeast Brazil (Kasahara and Yonenaga-Yassuda 1984). This paper compiles the karyotype of 271 species distributed throughout Brazil, representing an increase of more than 300%.

Since then, new cytotypes have been attributed to already known species. For instance, new diploid numbers were described for Ctenomys torquatus and new fundamental numbers for Oligoryzomys nigripes (described as Oryzomys nigripes – see references in Table 1). B chromosomes were described for Sooretamys angouya and also for four species of Dasyprocta. Undescribed rearrangements, including multiple sex chromosome system, were also detected (see Table 1). Moreover, new karyotypes that could not be correlated to any name were published, evidencing the possibility that an undescribed species may exist (*e.g.*: Akodon sp. n., Deltamys sp., Thaptomys sp., Euryoryzomys sp., Neacomys sp., Oecomys sp. 1 – 4, Oligoryzomys sp., Juliomys sp., Dasyprocta sp. Proechimys sp. – see Table 1). Additionally (as we will mention below) there are many species with a different diploid number associated that do not represent polymorphisms, which need to be revised (e.g. Euryoryzomys lamia, Euryoryzomys macconnelli, Hylaeamys yunganus, Oecomys auyantepui, Oecomys cleberi, Oecomys paricola, Oecomys roberti, Zygodontomys brevicauda, Rhipidomys nitela, Phyllomys pattoni, Proechimys guyannensis, etc.).

Since 1984, many species’ names have been redescribed or validated (e.g. Zygodontomys lasiurus was named as Bolomys lasiurus for a long time, and nowadays is Necromys lasiurus – see synonyms of Table 1). Also, due to the progress of molecular biology during the 1990, associated to morphological information, the number of species described has increased exponentially. It is important to emphasize that molecular phylogeny hitherto has contributed to better understand the cryptic diversity of Brazilian rodents, recognizing monophyletic clades. For instance, new candidate species of Akodon (Silva and Yonenaga-Yassuda 1998a, Silva et al. 2006), Oecomys (Suárez-Villota et al. under revision), Oligoryzomys (Andrades-Miranda et al. 2001a, Miranda et al. 2008), Neacomys (Silva et al. 2015, present paper), Thaptomys (Ventura et al. 2004, 2010), etc. were recognized based on new karyotypes associated to the monophyly of the samples. Even new genera were described based on multidisciplinary approaches: Drymoreomys (Percequillo et al. 2011) and Calassomys (Pardiñas et al. 2014).

Technological advances with fluorescent *in situ* hybridization (developed at the end of 1980’s but more used during 2000’s to date), made it possible to characterize chromosome rearrangements more precisely.

In this paper, we provide a new fundamental number for an undescribed species of Neacomys. The karyotype presented here (FN = 66) is similar to the one described by Silva et al. (2015) with FN = 64, except that we found five biarmed pairs and the distribution of constitutive heterochromatin in autosomes was restricted to pericentric regions. We suggest that differences in fundamental numbers are due to pericentric inversions in a small pair, since C-banding evidenced constitutive heterochromatin at the pericentromeric regions, and the morphology of chromosomes was accurately defined. Sex chromosomes presented the same morphology, although the Y was heterochromatic in the short arm (present paper), while it was entirely heterochromatic in the samples described by Silva et al. (2015).

Karyotype information was the first to point out that this specimen may represent a new species, since 2n = 58, FN = 66, has never been described for any Neacomys species. Although we used only one molecular marker (incomplete cyt-*b*), which was the same used by Silva et al. (2015), the phylogeny corroborates this information, since all samples with 2n = 58 clustered in a monophyletic high supported the clade. This included two well-supported structured clades, one with samples with FN = 70 (Chaves, Marajó Island) and the other with samples with FN = 64 and 66 (Marabá, Pará State and Vila Rica, Mato Grosso State, respectively), both sister clade to the sample from Igarapé-Açu, Amazonas State. Whether these samples belong to the same undescribed entity with strong population structure or whether they represent at least three different species must be clarified with further phylogeographic and morphological studies, including samples from other localities. This shows the importance of integrative approaches.

In fact, Neacomys have a greater diversity than previously known. Recently, based on morphology and molecular phylogeny, Hurtado and Pacheco (2017) demonstrated that Neacomys spinosus is a species complex and considered the subespecies Neacomys spinosus amoenus a valid species. After this revision, Neacomys spinosus is restricted to populations from Peruvian Amazon, and Neacomys amoenus encompasses two subspecies: Neacomys a. amoenus (from Brazilian Cerrado and Bolivia) and Neacomys a. carceleni (from Amazon basin of Ecuador, Brazil and Peru). Thus, sequences related to N. spinosus from central Brazil, and transition areas of Cerrado and Amazonia correspond to N. amoenus. Also, a new species, N. vargasllosai, from southern Peru and Bolivia was described. In this same revision, authors recovered three new species pending formal description (the first from Pará, Brazil, the second from Amazonas, Brazil, and the third from Peru and Ecuador). The one from Pará corresponds to the clade composed of samples with 2n = 58 (Fig. 2), reiteraiting the lack of knowledge in this genus.

The description of the karyotype of Carterodon sulcidens (a rare species) also corroborates the lack of knowledge for some species, and the importance of fieldwork in discovering new data.

We also show the picture of the karyotype of the exotic species Mus musculus for the first time. Despite the noteworthy variation in diploid numbers in Western Europe and Mediterranean populations because of Robertsonian rearrangements (Nachman et al. 1994), in Brazil, the only diploid number described was the standard one (2n = 40).

### Progress in cytogenetics: the molecular era

During the beginning of the 1970s (although banding techniques had already been described), karyotypes of Brazilian rodents were studied mainly through conventional staining and the description was limited to diploid and fundamental numbers. Even so, the idea of a wide chromosomal variability already existed. From the 1980s until now, comparative cytogenetics with chromosome banding persists and contributed for elucidating these variations, being that G and C-banding and Ag-NORs are the commonest and cheapest banding techniques.

In fact, the distribution of constitutive heterochromatin and Ag-NORs can be markers in some species. For example, large blocks of constitutive heterochromatin were detected in Clyomys laticeps (family Echimyidae) (Souza and Yonenaga-Yassuda 1984, Bezerra et al. 2012) and a huge heterochromatic arm in Pseudoryzomys simplex (family Cricetidae, subfamily Sigmodontinae, tribe Oryzomyini) (Moreira et al. 2013). C-band pattern is also an important technique for recognizing sex chromosomes, especially within the subfamily Sigmodontinae (Silva 1994, Di-Nizo 2013). Regarding the nucleolus organizer region, it seems that secondary constriction is a characteristic feature of the family Echimyidae and, as with other vertebrates, may be an important marker. However, chromosomal comparison is now passing from banding patterns to the use of higher resolution innovation of molecular cytogenetics using FISH.

FISH using chromosome painting allows a comparison in a wide genomic scale, revealing a greater number of chromosome changes, unrevealed by the commonest banding techniques, especially in the tribes Akodontini and Oryzomyini of the Subfamily Sigmodontinae. For instance, G-banding pattern showed several rearrangements between Akodon species (Tribe Akodontini) (Geise et al. 1998, Silva et al. 2006), but much more complex rearrangements within this genus were observed after cross-species chromosome painting (Ventura et al. 2009).

Extensive chromosomal rearrangements such as Robertsonian, *in tandem* fusion/fission and pericentric inversion, were also observed within the genus Oligoryzomys (Tribe Oryzomyini), after chromosome painting. Using a molecular phylogeny as a reference, it was also possible to detect the direction of the rearrangements and to infer that fission events were as common as fusion events (Di-Nizo et al. 2015). Moreover, Robertsonian rearrangement between Oryzomys rupestris Weksler & Bonvicino, 2005 (referred as Oligoryzomys sp. 1), 2n = 46, FN = 52, and Oligoryzomys sp. 2, 2n = 46, FN = 52 was firstly detected by using classic cytogenetic and FISH with telomeric probes (Silva and Yonenaga-Yassuda 1997) and later corroborated by chromosome painting (Di-Nizo et al. 2015). However further studies with molecular phylogeny and morphology are necessary to clarify if both entities represent a single species (with a polymorphism spread in the population) or two different species (in the case of this rearrangement resulted in reproductive incompatibilities leading to the speciation of ancestral population).

The advent of chromosome painting made it possible to compare not only related species but also distant ones, something which is difficult to achieve with banding patterns. Hass et al. (2008) compared Mus musculus (family Muridae) to Akodon species (family Cricetidae); Nagamachi et al. (2013) compared two different, unrelated genera of the tribe Oryzomiyni (Cerradomys and Hylaeamys) and Suárez et al. (2015) and Pereira et al. (2016) compared homologies between the tribes Akodontini and Oryzomyini.

Despite the ‘modern cytogenetics era’, chromosome banding is still an important tool for animal cytogenetic studies, not only because FISH cannot reveal chromosome inversions, but also because it is still a difficult and expensive technique to use.

### Chromosome rearrangements and speciation

Rodents proved to be a good model for chromosome evolution studies. Cytogenetics associated with molecular or morphological phylogenetic reconstruction broke cytogeneticist paradigms that fusion rearrangement is more common than fission, and that the reduction in 2n is the expected pattern (e.g. Di-Nizo et al. 2015).

Chromosomal rearrangement could possibly be the cause of reproductive isolation in many Brazilian rodents’ species, leading to speciation. The main rearrangements that lead to species formation are Robertsonian, *in tandem* fusion/fission and pericentric inversion, while the variability in constitutive heterochromatin does not seem to create a reproductive barrier and consequent speciation (King 1993, Romanenko and Voloboeuv 2012).

For a long time, it was thought that chromosomal structural rearrangements promoted speciation by generating gametes with duplications and deficiencies, therefore, causing less adaptability of the heterozygotes, but this model was rejected because it lacked theoretical support (Rieseberg 2001, Patton 2004, Jackson 2011). Recently, a different model of chromosome speciation was proposed in which the gene flow is reduced because of recombination-suppression in rearranged regions (Noor et al. 2001, Rieseberg 2001).

In fact, normal meiotic behavior with suppression of crossing over in inverted segments of heteromorphic chromosomes caused by pericentric inversions of Akodon cursor and Oligoryzomys nigripes was observed, with non-selective disadvantages in heterozygous carries (Fagundes et al. 1998, Bonvicino et al. 2001a). Some genetic mechanisms seem to be responsible for overcoming meiotic errors in heterozygous individuals, such as the occurrence of heterosynapsis and the low frequency of chiasm between the inverted segments.

A remarkable chromosome variation can be found in the semi- and fossorial Brazilian rodents Blarinomys breviceps (in which molecular phylogeny demonstrated two structured clades – see Ventura et al. 2012), Clyomys laticeps and Ctenomys minutus. Their species status, and whether their chromosome variation is adaptative and correlated with ecological patterns should be evaluated.

For example, a very well-known case of chromosome speciation due to population adaptation to climatic stress and ecological unpredictability was described in the subterranean rodent Spalax ehrenbergi (Family Spalacidae) found in Israel, in which diploid numbers increase coincidently with geographic regions of high aridity (Wahrman et al. 1969). The weak dispersion pattern of this fossorial rodent may have contributed to the fixation of adaptative chromosome change (Árnason 1972).

### Cytotaxonomy

Cytotaxonomy is the use of chromosome data as the first clue in the identification of species. Since many Brazilian rodent species present species-specific karyotype and show morphological similarities, chromosome information showed to be useful in the diagnosis of species.

The present revision showed that the delimitation of species based on chromosome data (cytotaxonomy) is essential for recognizing some species of the genera Akodon, Calomys, Cerradomys, Euryoryzomys, Delomys, Hylaeamys, Juliomys, Neacomys, Oecomys, Oligoryzomys (family Cricetidae, subfamily Sigmodontinae), Ctenomys (family Ctenomyidae), and Thrichomys and Trinomys (family Echimyidae).

On the other hand, since rates of karyotype evolution differ in distinct branches of the rodents’ phylogeny, some species present identical diploid and fundamental numbers, and they cannot be identified solely through chromosome data. This is the case of the following species: (i) Cavia aperea, Cavia fulgida and Cavia magna; (ii) Kerodon acrobata and Kerodon rupestris (Family Caviidae); (iii) Akodon lindberghi and A. mystax; (iv) Akodon paranaensis and A. reigi; (v) Brucepattersonius griserufescens, B. iheringi, B. soricinus and Thaptomys nigrita; (vi) Oxymycterus caparoae, Oxymycterus dasytrichus, Oxymycterus nasutus and Oxymycterus roberti (the other four species of Oxymycterus also have the same diploid number but lacks information on FN) (Family Cricetidae, Subfamily Sigmodontinae, Tribe Akodontini); (vii) Cerradomys marinhus and Pseudoryzomys simplex; (viii) Drymoreomys albimaculatus and Oecomys sp. 4; (vix) Euryoryzomys emmonsae, E. nitidus and E. russatus (despite E. nitidus and E. russatus have disjunction distribution); (x) Holochilus brasiliensis and Nectomys squamipes; (xi) Hylaeamys laticeps and Hylaeamys seuanezi; (xii) Hylaeamys oniscus and H. perenensis; (xiii) Oecomys bahiensis, Oecomys concolor, Oecomys sp. 2 and sp. 3; (xiv) Neacomys dubosti and N. amoenus (family Cricetidae, Subfamily Sigmodontinae, tribe Oryzomyini); (xv) Rhipidomys cariri, R. gardneri, R. tribei, R. itoan and R. macconnelli (family Cricetidae, Subfamily Sigmodontinae, Tribe Thomasomyini); (xvi) Dasyprocta azarae, D. iacki, D. fuliginosa, D. leporina, D. prymnolopha, D. variegata and Dasyprocta sp. (family Dasyproctidae); (xvii) Isothrix bistriata, Mesomys hispidus, M. stimulax, Trinomys albispinus and T. dimidiatus; (xviii) Proechimys brevicauda and Proechimys cuvieri; (xix) Proechimys gardneri and Proechimys pattoni (family Echimyidae) and (xx) Guerlinguetus brasiliensis and Hadrosciurus spadiceus (family Sciuridae) (Table 1).

Furthermore, some unrelated species, that belong to different tribes, or even families, present the same diploid and fundamental number, suggesting a homoplastic character: (i) Hylaeamys megacephalus and Oxymycterus delator; (ii) Juliomys pictipes and Thalpomys cerradensis; (iii) Calomys laucha and Neacomys amoenus (although there are differences in the size of the biarmed chromosomes); (iv) Oecomys franciscorum and Delomys sublineatus (despite the first acrocentric pair in D. sublineatus is bigger than in Oryzomys franciscorum as well as the biarmed pair in the last species); (v) Coendou melanurus and Oligoryzomys utiaritensis; (vi) Ctenomys ibicuiensis and Scolomys ucayalensis and (vii) Callistomys pictus, Coendou spinosus and Myocastor coypus.

### Interdisciplinarity

Since the beginning of the cytogenetic studies in Brazilian rodents, there have been cases in which different karyotypes were assigned to one species or the same karyotype was referred to in different species. In fact, many of these cases were solved after the integration of different disciplines. For instance, for many years cytogenetic information indicated that the previous “Oryzomys subflavus” could, in fact, be more than one species, since nine different karyotypes were attributed to a single taxonomic entity (Maia and Hulak 1981, Almeida and Yonenaga-Yassuda 1985, Svartman and Almeida 1992, Silva 1994). Nowadays, after interdisciplinary studies with morphology and molecular phylogeny, it is possible to recognize eight species (Weksler et al. 2006, Percequillo et al. 2008, Tavares et al. 2011, Bonvicino et al. 2014). Moreover, for a long time Nectomys was represented by only one species in Brazil, with two diploid numbers (2n = 52 + 1 to 3 Bs and 2n = 56 + 1 to 3 Bs). Nevertheless analyses of the spermatogenesis in hybrids and the sterility of crosses between both cytotypes indicated that Nectomys should be considered two distinct species: Nectomys rattus (2n = 52) and Nectomys squamipes (2n = 56) (Bonvicino et al. 1996).

The opposite occurred in the genus Oligoryzomys since the same karyotype (2n = 62, FN = 80-82) was attributed to different names (Oryzomys nigripes, Oryzomys delticola, and Oryzomys eliurus). After molecular and morphology integration, Oryzomys delticola and Oryzomys eliurus were considered as a junior synonym of Oryzomys nigripes (Bonvicino and Weksler 1998).

Some of these cases persist until today, for instance, more than one karyotype was described for Euryoryzomys macconnelli and E. lamia (Table 1). Molecular phylogeny and morphology corroborate the species complex status of both entities (Almeida 2014, Percequillo 2015a). Similarly, Oecomys roberti, Oryzomys paricola, and Oryzomys catherinae are probably species complexes, not only because of their variability in diploid number, but also because of phylogenetic reconstruction and morphological studies (Suárez-Villota et al. 2017). Ctenomys minutus, C. torquatus, Hylaeamys yunganus, Rhipidomys nitela, Sigmodon alstoni and Zygodontomys brevicauda also deserve taxonomic attention because they may represent cases in which different diploid numbers are attributed to the same names. Similarly, Blarinomys breviceps has a variable diploid number and two geographic structured clades were recovered in the molecular phylogeny (Ventura et al. 2012), indicating that a morphological revision is needed.

Remarkably, such examples can also be found in the family Echimyidae. The need to use different approaches for taxonomic revision is clear in order to investigate whether Phyllomys blainvillii, Phyllomys pattoni, and Proechimys guyannensis represent species complexes, given the fact that they have more than one karyotype associated.

Interdisciplinary approaches, including cytogenetic, molecular phylogeny, morphology and geographic distribution are essential for accessing the limits of Brazilian rodents’ species. One of the best-known examples was the old genera Oryzomys, considered the most complex and composing almost half of the species of the tribe Oryzomyini (Musser and Carleton 1993). The current genera Melanomys, Microryzomys, Nesoryzomys, Oecomys, and Oligoryzomys, were first considered a subgenus of Oryzomys and later elevated to the category of genus after morphology, chromosomal and molecular analyses (Myers et al. 1995, Smith and Patton 1999, Bonvicino and Moreira 2001). Another outstanding example of an integrative approach was the study in which ten new genera were described for species that were previously referred to as Oryzomys (Weksler et al. 2006), corroborating the cryptic diversity in Oryzomyini previously indicated by cytogenetic data.

Within the Family Echimyidae, the association of morphology and molecular analysis was essential for elevating Trinomys (considered subgenus of Proechimys) to the genus category (Lara et al. 1996, Leite and Patton 2002).

### Perspectives

Despite the new technological approaches, chromosome characterization with conventional staining and banding pattern is still important, mainly because 38 species lack any karyotype information (Table 1). From this amount, 16 are distributed in the Amazonian biome, evidencing the lack of knowledge for this region. The fieldwork is very important and must be encouraged not only because new species and even genera are constantly being described but also because cytogenetic and distribution information of several species are poorly known.

Concerning the family Echimyidae, it is noteworthy that cytogenetic information is lacking for more than 20% of its species. Eleven out of 17 echimyid genera which occur in Brazil are arboreal (Galewski et al. 2005, Emmons et al. 2015). The issues for sampling small arboreal mammals and the consequent low number of studies with this approach have already been highlighted in the literature (Malcolm 1991, Taylor and Lowman 1996, Graipel et al. 2003). In this sense, it can be inferred that this deficiency in echimyid cytogenetic knowledge may be related to sampling scarcity.

The future of molecular biology is promising, with next-generation sequencing (NGS) technology and mitogenomics hopefully providing more robust phylogenetic studies. This new approach was performed with the Family Echymyidae, revealing new supported nodes and clarifying some aspects of the group’s taxonomy (Fabre et al. 2016).

However, it is important to reiterate the heterogeneity of characters since DNA, chromosomes, morphology, and behavior are not evolving at the same rate. This particularity may imply in different taxonomic interpretations, with a population being identified as a unique species by one character and two or more species by another, especially in the cases of recent or ongoing speciation. The consequences can be taxonomic inflation or underestimation of the biodiversity, and that is why interdisciplinary approaches are crucial to better understand the biological diversity of rodents.

## Supplementary materials

Supplementary material 1 Table S1 Data type: molecular data Explanation note: Sequences analysed for phylogenetic reconstruction (Maximum likelihood and Bayesian Inference) of Neacomys, with species, GenBank and lab/ field number, diploid and fundamental number (when available), locality and reference. Abbreviations: N/A means that the information is not available. *Cytogenetic data analysed in this work. In bold, sequences obtained in this work. Coordinates for Neacomys specimens studied herein: Amazonas State: Igarapé-Açu (04°20'S, 58°38'W); Mato Grosso State: Aripuanã (10°10'S, 59°27"W); Cláudia (11°30'S, 54°53'W); Vila Rica (09°54'S, 51°12'W). Museum and collector acronyms for specimens studied herein: APC (Ana Paula Carmignotto), CIT (Laboratório de Citogenética de Vertebrados - IBUSP), MTR (Miguel Trefaut Rodrigues), MZUSP (Museu de Zoologia, Universidade de São Paulo, Brazil) and PEU (Pedro Luís Bernardo da Rocha). This is an open access article distributed under the terms of the Creative Commons Attribution License (CC BY 4.0), which permits unrestricted use, distribution, and reproduction in any medium, provided the original author and source are credited.Camilla Bruno Di-Nizo, Karina Rodrigues da Silva Banci, Yukie Sato Kuwabara, Maria José de J. Silva
